# Evaluation of Functional Recovery in Rats After Median Nerve Resection and Autograft Repair Using Computerized Gait Analysis

**DOI:** 10.3389/fnins.2020.593545

**Published:** 2021-01-21

**Authors:** Johannes C. Heinzel, Viola Oberhauser, Claudia Keibl, Nicole Swiadek, Gregor Längle, Helen Frick, Jonas Kolbenschlag, Cosima Prahm, Johannes Grillari, David Hercher

**Affiliations:** ^1^Department of Hand, Plastic, Reconstructive and Burn Surgery, BG Trauma Center Tuebingen, Eberhard Karls University Tuebingen, Tuebingen, Germany; ^2^Ludwig Boltzmann Institute for Experimental and Clinical Traumatology, Vienna, Austria; ^3^Austrian Cluster for Tissue Regeneration, Vienna, Austria; ^4^Department of Biotechnology, Institute of Molecular Biotechnology, BOKU–University of Natural Resources and Life Sciences Vienna, Vienna, Austria

**Keywords:** gait analysis, functional recovery, grasping strength, median nerve, rats, catwalk, nerve repair, microsurgery

## Abstract

Computerized gait analysis is a common evaluation method in rat models of hind limb nerve injuries, but its use remains unpublished in models of segmental nerve injury of the forelimb. It was the aim of this work to investigate if computerized gait analysis is a feasible evaluation method in a rat model of segmental median nerve injury and autograft repair. Ten male Lewis rats underwent 7-mm resection of the right median nerve with immediate autograft repair. The left median nerve was resected without repair and served as an internal control. Animals were assessed for 12 weeks after surgery *via* CatWalk (CW) gait analysis every 2 weeks. Evaluation of motor recovery by means of the grasping test was performed weekly while electrophysiological measurements were performed at the end of the observation period. CW data were correlated with grasping strength at each post-operative time point. CW data were also correlated with electrophysiology using linear regression analysis. Principal component analysis was performed to identify clusters of outcome metrics. Recovery of motor function was observable 4 weeks after surgery, but grasping strength was significantly reduced (*p* < 0.01) compared to baseline values until post-operative week 6. In terms of sensory recovery, the pain-related parameter Duty Cycle showed significant (*p* < 0.05) recovery starting from post-operative week 8. The Print Area of the right paw was significantly (*p* < 0.05) increased compared to the left side starting from post-operative week 10. Various parameters of gait correlated significantly (*p* < 0.05) with mean and maximum grasping strength. However, only Stand Index showed a significant correlation with compound muscle action potential (CMAP) amplitude (*p* < 0.05). With this work, we prove that computerized gait analysis is a valid and feasible method to evaluate functional recovery after autograft repair of the rat median nerve. We were able to identify parameters such as Print Area, Duty Cycle, and Stand Index, which allow assessment of nerve regeneration. The course of these parameters following nerve resection without repair was also assessed. Additionally, external paw rotation was identified as a valid parameter to evaluate motor reinnervation. In summary, computerized gait analysis is a valuable additional tool to study nerve regeneration in rats with median nerve injury.

## Introduction

In experimental research on peripheral nerve injury, the sciatic nerve model is the most frequently used (Tos et al., 2009; Vela et al., 2020). Within the broad range of evaluation methods to determine experimental outcome, e.g., histology, electrophysiology, or retrograde labeling, the assessment of functional recovery is the most important criterion (de Medinaceli et al., 1982). However, the sciatic nerve model has some major disadvantages, such as its mixed fiber type and innervation of antagonistic muscles (Stromberg and Hebel, 1976). These strongly limit the maximum degree of functional recovery after injuries in which the continuity of the nerve's fascicular structure has been disrupted (Haastert-Talini, 2017). Additionally, automutilation is a common problem in rats experiencing neuropathic pain after sciatic nerve injury (Irintchev, 2011). This results in amputation of the toes or parts of the paw and in consequence only limited or even impossible functional evaluation (Weber et al., 1993). Besides other emerging alternatives to the sciatic nerve model, e.g., the femoral nerve model (Hercher et al., 2019; Heinzel et al., 2020b), the median nerve model is gaining increasing attention within the scientific community (Ronchi et al., 2019). In rats, as in humans, the median nerve is one of the main nerves of the brachial plexus, originating from the spinal roots C_5_ to Th_1_. It provides muscular innervation to the flexor muscles of the front limb and paw. The median nerve also provides sensory fibers to the medial aspect of the paw and digits I–III (Jackson, 1936; Bertelli et al., 1995). An advantage of this model is its translational potential given the predominance of upper limb nerve injuries in human patients (Foster et al., 2019; Karsy et al., 2019). Additionally, although neuropathic pain is induced after median nerve injury (Lue et al., 2002), cases of automutilation are extremely rare in this model (Papalia et al., 2003). A plethora of evaluation methods is used for the assessment of functional recovery after median nerve injury (Stossel et al., 2017), of which the grasping test is the predominant one (Bertelli and Mira, 1995; Hanwright et al., 2019). In rats, unlike in humans, the superficial and deep finger flexor muscles are predominantly innervated by the median nerve. Therefore, rats lose the ability to flex their digits when the nerve is transected, which is assessable *via* the grasping test (Tos et al., 2009). Evaluation of functional recovery *via* the gait analysis, either by means of ink and paper or by computerized approaches, is among the most commonly used assessments to evaluate recovery of locomotor function after sciatic nerve axonotmesis, constriction, or neurotmesis (Navarro, 2016). Among other approaches, such as the GaitScan, DigiGait^Tm^, or TreadScan^Tm^, the CatWalk (CW) method is an automated gait analysis system. It uses footprint illumination to quantify gait behavior in rats and mice (Heinzel et al., 2020a). The method allows for evaluation of a plethora of parameters related to gait, the ones routinely and most assessed in rats with peripheral nerve injury are listed in Table 1. Some parameters have been shown to be related to pain, although this does not imply that they cannot also be altered by impaired motor or sensory function in general (Vrinten and Hamers, 2003; Heinzel et al., 2020b). Other parameters have been subsumed under the term general parameters of gait or coordination-related parameters of gait. The latter have been shown to be affected by central nervous system pathologies, such as spinal cord injuries (Deumens et al., 2007). For this work, we adapted this pre-existing classification system and expanded it further by means of a fourth category: other parameters of gait, which includes all parameters not attributable to one of the other three aforementioned categories (Table 1). Gait analysis is used rarely in this context, and only four studies could be identified that made use of it in rat models of median nerve injury. Wang et al. (2008) studied locomotor behavior after either isolated or combined median, ulnar, and radial nerve injury with immediate repair *via* two-dimensional digital video motion analysis. The authors were able to show that flexion of both wrist and metacarpophalangeal joints was decreased after median nerve injury. In addition, linear regression revealed a strong correlation between the joint angles and grip strength measurements (Wang et al., 2008). Casal et al. (2018) used walking track analysis by staining the rats' forepaws with methylene blue and letting them walk through a wooden corridor whose floor was paved with graph paper. Besides measuring the dimensions of the paw prints, such as the area of the paw impression and its maximum length and width, the authors also assessed Stride Length and Base of Support (BoS) before and after resection of a 10-mm segment of the rat median nerve with immediate autograft repair (Casal et al., 2018). The CW device was used in only two studies, both conducted by Chen et al. (2012a,b). In their work, the authors combined a 2-mm resection and ligation of the median nerve to investigate the sequela of neuropathic pain *via* immunohistochemical and assessment of gait (Chen et al., 2012a,b). These studies revealed that changes of gait can be monitored by the CW parameters of Print Length, Print Width, Print Area, and Mean Paw Print Intensity. Although rats were only observed for 28 days and the nerve was not surgically reconstructed, these studies' results indicated the potential of the evaluation method in the median nerve neurotmesis model. In order to allow testing of new treatment strategies after peripheral nerve injury, a reliable preclinical animal model is necessary. The median nerve model is well-established for this purpose (Ronchi et al., 2019). However, to the best of our knowledge, the CW device has never been used to evaluate locomotor function after segmental median nerve injury and repair. It was the aim of this work to investigate whether automated gait analysis is a feasible method to evaluate functional recovery after median nerve resection and autograft repair. We further aimed to perform an assessment of gait after nerve resection without subsequent autograft repair to provide additional information regarding the impact of median nerve injury and reconstruction on functional recovery.

**Table 1:** Parameters of gait assessed in this study.

***Category*** **I: General** **II: Pain-related** **III: Coordination-related** **IV: Other**	***Parameter***	***Explanation***
I	Print Length (distance unit)	Length of the paw print
I	Print Width (distance unit)	Width of the paw print
I	Print Area (distance unit)	Area of the paw print
I	Base of Support (BoS) (distance unit)	Distance between the two hind-or front paws
I	Stride Length (distance unit)	Distance between two consecutive placements of a paw
II	Mean Paw Print Intensity (arbitrary units)	Mean intensity of the paw
II	Swing Time (seconds)	Swing Time of the paw
II	Stand Time (seconds)	Stand Time of the paw
II	Duty Cycle (%)	Stand Time divided by Stand Time plus Swing Time
III	Normal Step Sequence Patterns (NSSP)	Specific sequences of paw placements during a step cycle
III	Phase Dispersions / Phase Lags/ Couplings (%)	Temporal differences between the step cycles of two paws
III	Regularity Index (RI) (%)	Quantification of interlimb coordination
IV	Swing Speed (centimetres / second)	Swing Speed of the paw
IV	Stand Index (%)	Measurement for the speed with which the paw loses contact with the ground

*Note that the table does not feature all parameters that are assessable via CatWalk (CW) but only the ones that are routinely studied in experimental rodent models of peripheral nerve injury (PNI)*.

## Materials and Methods

### Ethical Approval

The experimental protocol was approved in advance by the Animal Protocol Review Board of the City Government of Vienna. All procedures were carried out in full accord with the Helsinki Declaration on Animal Rights and the Guide for the Care and Use of Laboratory Animals of the National Institutes of Health.

### Animals and Surgery

Ten male Lewis rats (Janvier Labs, Le Genest-Saint-Isle, France), weighing 280–350 g, were kept in groups of two or three in appropriate cages according to internal standard operating procedures. The animals had access to food and water *ad libitum*. After the rats were allowed to get accustomed to their new surrounding for 7 days prior to any experimental handling and after completing a 7-day training period on the CW device, they underwent bilateral surgery of the median nerve under an operation microscope (Leica M651, Leica Microsystems, Vienna, Austria). A 7-mm segment of the left and right nerve was removed microsurgically by performing a transection about 1.5 mm proximal to the position where it is crossed over by the brachial artery and vein and another transection 7 mm proximal to the first one. On the right side, the gap was bridged with the original nerve segment in reverse fashion as a homotopic nerve autograft with two sutures per coaptation site (Ethilon® 10-0, Ethicon-Johnson & Johnson, Brussels, Belgium). On the left side, the nerve defect remained unreconstructed to serve as an internal control group. To prevent spontaneous regeneration, the distal nerve stump was sutured into the short head of the biceps muscle. The post-operative observation period lasted 12 weeks (*n* = 10).

### Functional Analysis

#### CatWalk XT

Functional analysis was performed using the CatWalk XT (Version 10.6) automated gait analysis system (Noldus Information Technology, Wageningen, The Netherlands). The system's main component is a walkway with a glass floor, illuminated by a green walkway and red ceiling light sources. Whenever a rodent crosses the glass plate, the animal's paw prints get illuminated by the green light source while the red ceiling light's purpose is to provide contrast for the animal's body contour. All images are recorded by a fully automated camera mounted underneath the glass plate and processed by the system's software. The software then calculates a multitude of static and dynamic parameters of gait (Table 1). Sufficient habituation to the testing procedure is necessary since the animals need to learn to cross the walkway without interruption and with uniform walking speed. These points have been stressed as paramount prerequisites for valid data acquisition (Deumens et al., 2014). The animals were trained daily to cross the walkway for 1 week before performing baseline evaluation and surgery. They were examined post-operatively in intervals of 13–15 days. Rats were trained to cross the walking track with a speed between 50 and 100 cm/s, in accordance with published literature (Koopmans et al., 2007). We collected three runs per trial until 12 weeks after surgery. Animals were rewarded with 1–2 pellets of cereal after completion of each data acquisition session, but no food deprivation was used beforehand. Data were collected according to the established standards as recommended by other authors (Koopmans et al., 2007; Deumens et al., 2014). The following 14 parameters were assessed (Table 1): BoS (mm), Stride Length (mm), Print Length (mm), Print Width (mm), Print Area (mm^2^), Swing Speed (s), Stand Time (s), Duty Cycle (%), Stand Index (%), Swing Time (s), Regularity Index (RI) (%), distribution of Normal Step Sequence Patterns (NSSPs) (%), Phase Dispersions (%), and Paw Angle Body Axis (PABA, the angle between each front paw and the body axis of the animal) (°). All parameters with the exception of the BoS and the last four were assessed for all paws, and for each one, a ratio was calculated by dividing the right side's value (resection and homologous autograft repair) by either the left front limb's value (negative control) or the right hind limb's value (positive control). This ratio was then compared to the ratio at baseline for each postsurgical time point, and the result was given in percent. All data in this work were calculated and graphed as mean ± standard error of the mean (SEM).

#### Reflex-Based Grasping

For the assessment of grasping strength, rats were evaluated biweekly *via* the grasping test until 12 weeks after surgery (Bertelli and Mira, 1995). This method, which was first introduced by Bertelli and Mira in 1995 and further refined in the following years, allows evaluation of finger flexor function by means of a simple testing apparatus, e.g., a grid or bar that is connected to a scale (Papalia et al., 2003; Tos et al., 2009; Daeschler et al., 2018a). Grasping strength is mainly dependent on the flexion force exerted by the distal phalanges, which is mediated by the long finger flexor muscles. In the rat, the long finger flexors are predominantly innervated by the median nerve. Therefore, this nerve's function can easily be assessed when grasping strength is exerted by the long finger flexor muscles only, e.g., under elimination of the wrist and elbow flexor muscles. For this purpose, a Grip-Strength Meter (Ugo Basile S.R.L., Gemonio V.A., Italy) was mounted in a vertical position. The rats were gently lowered by holding them at the tail until they reached for the grasping bar. By placing a band of adhesive tape just a few millimeters underneath the bar, the rats were prevented to encompass the bar with their wrist to prevent usage of the wrist flexors. The animals were then gently lifted upward until they lost grip. Only trials without elbow flexion, e.g., usage of the elbow flexor muscles, were considered valid. We recorded three valid trials per rat at each time point, and the maximum force in grams was recorded for each trial. The mean grasping force was then calculated by averaging the maximum force of all three trials. Additionally, grasping ability was scored by means of a trinary scale as suggested by Stossel et al. (2017). In brief, missing finger flexion was scored as 1/3, while finger flexion without measurable force was scored 2/3. Finger flexion with measurable force was scored 3/3.

#### Electrophysiological Analysis

To evaluate successful reinnervation of the flexor digitorum superficialis (FDS) muscle, latency and peak amplitude (hereafter referred to as amplitude) of the muscle's compound muscle action potential (CMAP) were measured at the end of the observation time at 12 weeks (WPO12) in all 10 animals. The median nerve was explored, and for stimulation of the motor branch, a bipolar stimulation electrode was placed proximal to the nerve's ramifications in the cubital fossa using a micro manipulator. Two needle electrodes were placed into the FDS muscle ~20 mm apart for recording, whereas the grounding electrode was placed in the subcutaneous tissue of the right hind limb. A Neuromax EMG device (Natus, WI, United States) was used for stimulation and recording. Core temperature of the animal was measured rectally and used for normalization.

### Statistical Analysis

All statistical analyses were performed using IBM SPSS Version 26 (International Business Machines Corporation, Armonk, NY, USA. For the CW and grip strength data, means of all parameters were tested for statistically significant differences at each measured time point using repeated measures ANOVA (RMANOVA). The Bonferroni correction was used to adjust the level of significance, and *p*-values of 0.05 or lower were considered statistically significant. For the analysis of correlation between CW data and electrophysiological data, Print Area, Print Length, Print Width, Swing Speed, BoS, Duty Cycle, Swing Time, and Mean Paw Intensity were correlated to both peak amplitude and CMAP at WPO12 using linear regression analysis with IBM SPSS Version 24. We decided to perform regression analysis with the general and pain-related parameters only. As the coordination-related parameters of RI, NSSP, and Phase Dispersions are related to all four paws, correlating them with electrophysiological measurements of one paw alone would in our opinion be of reduced validity. *P*-values of 0.05 or lower were considered statistically significant. For the analysis of correlation between the CW data and grip strength, the previously mentioned CW data were correlated to the measured grasping force at all time points of concomitant data acquisition. Pearson correlation coefficient and *p*-value (two-tailed) were calculated. Multivariate analysis of gait parameters and grasping strength was performed by means of the principal component analysis (PCA) method. The scree plot method was used to determine the number of principal components to be included. Further analysis with direct Oblimin rotation was used to extract principal component clusters (Jackson, 2005; David and Jacobs, 2014; Ledesma et al., 2015). A loading magnitude of >0.4 was considered significant (Stevens, 1996).

## Results

### Animal Welfare

All animals recovered well from surgery. There were no cases of limb automutilation or contractures during the observation period. One animal's data had to be excluded from the analysis of electrophysiological evaluations due to technical problems during the measurements.

### CatWalk XT Gait Analysis

**Figure 1:**
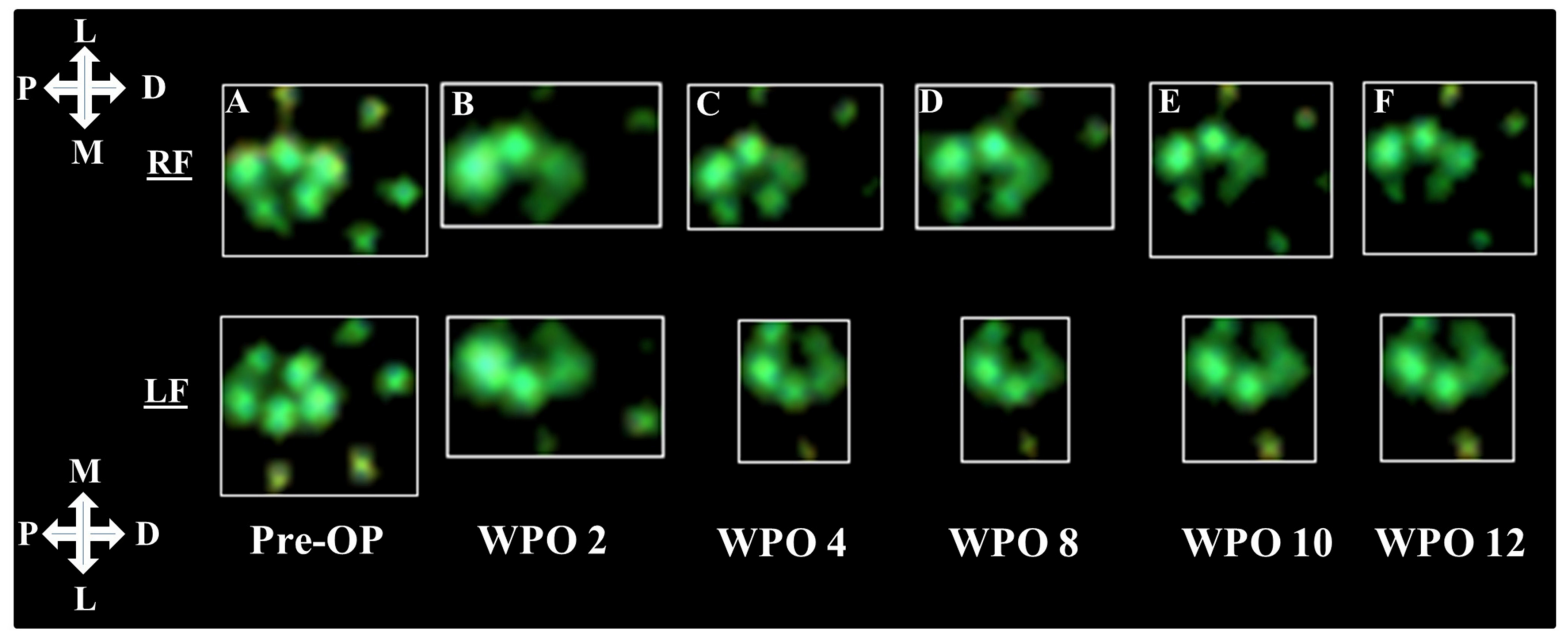
Representative results of paw prints as assessed *via* CatWalk (CW) gait analysis (Software Version 10.6). Paw prints of both the right and left front paws are shown starting from Pre-OP measurements **(A)** and over the course of the 12-week observation period **(B–F)**. The white crosses indicate the orientation of the respective paw in relation to the rat's body axis; P, proximal; D, distal; M, medial; L, lateral.

Representative results of the rats' paw prints prior to and following 7-mm median nerve resection and autograft repair are shown in Figure 1. At WPO2 (Figure 1B), the animals mainly used the lateral half of both front paws including the two lateral toes for weight support. Both medial toes were either completely undetectable or only faintly visible during CW data acquisition. While weight support on the medial half of the right front paw (RF) increased from WPO4 (Figure 1C) until WPO12 (Figure 1F), paw prints of the left front paw (LF) remained unchanged between WPO4 and WPO12 with only the most lateral toe visible during data acquisition. Interestingly, the third toe of LF (second most lateral) was visible at WPO4, indicating weight support on this part of the paw at this particular time point. However, in the following weeks until the end of the observation period, this was no longer observable.

#### General Parameters of Gait

##### Print Area

**Figure 2:**
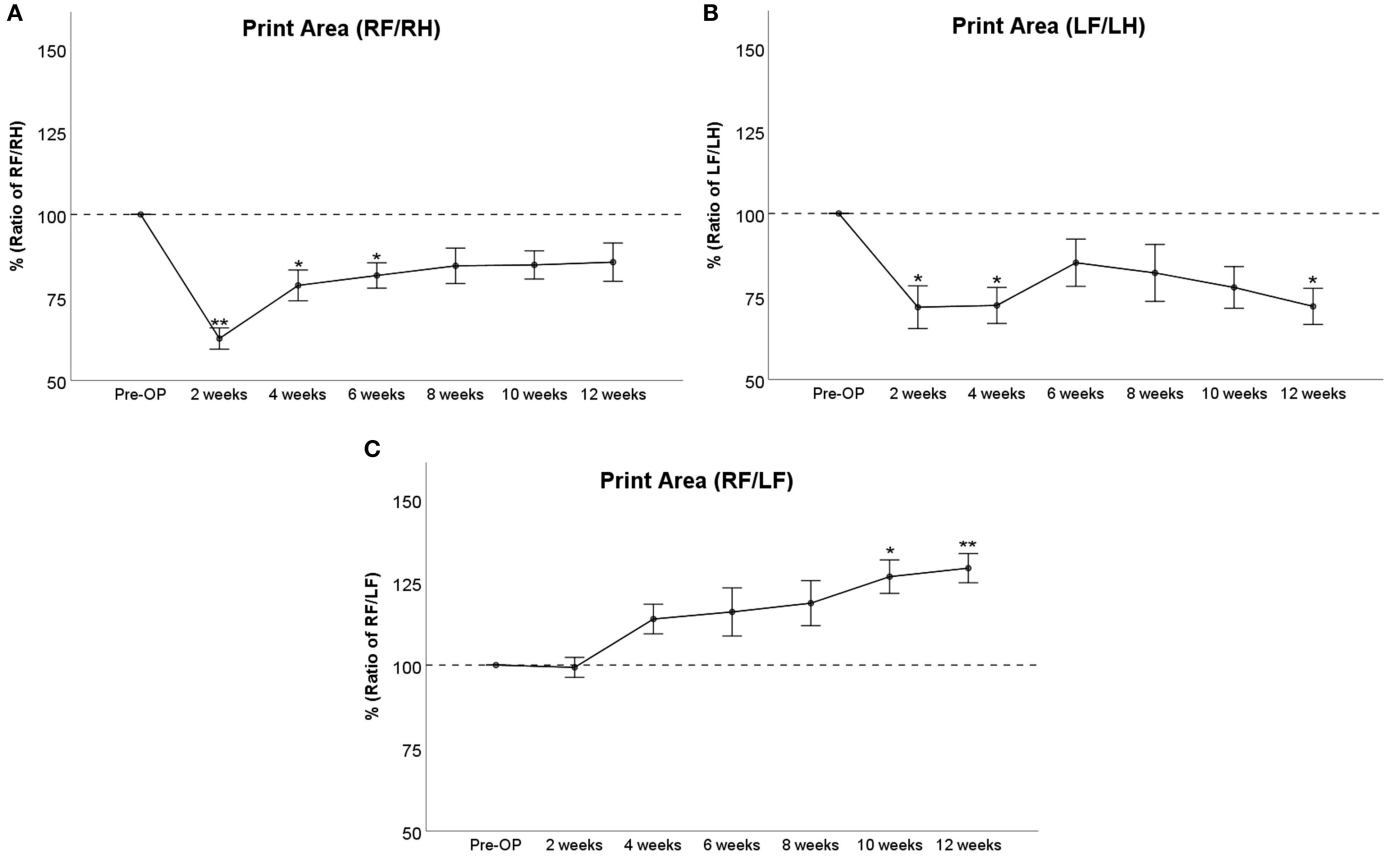
Course of Print Area either as a ratio of the reconstructed right front paw (RF) and the uninjured right hind paw (RH) **(A)**, the non-reconstructed left front paw (LF) and the uninjured left hind paw (LH) **(B)** or as ratio of both front paws **(C)** following bilateral median nerve neurotmesis and unilateral autograft repair, *p < 0.05 as compared to Pre-OP, **p < 0.01 as compared to Pre-OP.

At WPO2, right front/right hind (RF/RH) Print Area (Figure 2A) was highly significantly (*p* < 0.01) reduced to <65% compared to baseline values. It steadily increased thereafter to 79% at WPO4 and 81% at WPO6 and reached 85% of baseline at WPO12. This was not significantly different from baseline values starting from WPO8. Left front/left hind (LF/LH) Print Area (Figure 2B) was significantly (*p* < 0.05) reduced to 72% of baseline at WPO2 and WPO4. It accounted for 85, 82, and 78% at WPO6, WPO8, and WPO10, respectively, which was not significantly reduced compared to Pre-OP values. At WPO12, values decreased to 72% again, which was significant (*p* < 0.05) compared to baseline measurements. RF/LF (reconstructed side/unreconstructed side) Print Area (Figure 2C) started to increase from WPO4 and reached 119% of Pre-OP values at WPO8. The parameter was significantly increased to 126% of baseline at WPO10 (*p* < 0.05) and to 129% of baseline at WPO12 (*p* < 0.01).

##### Print Length

**Figure 3:**
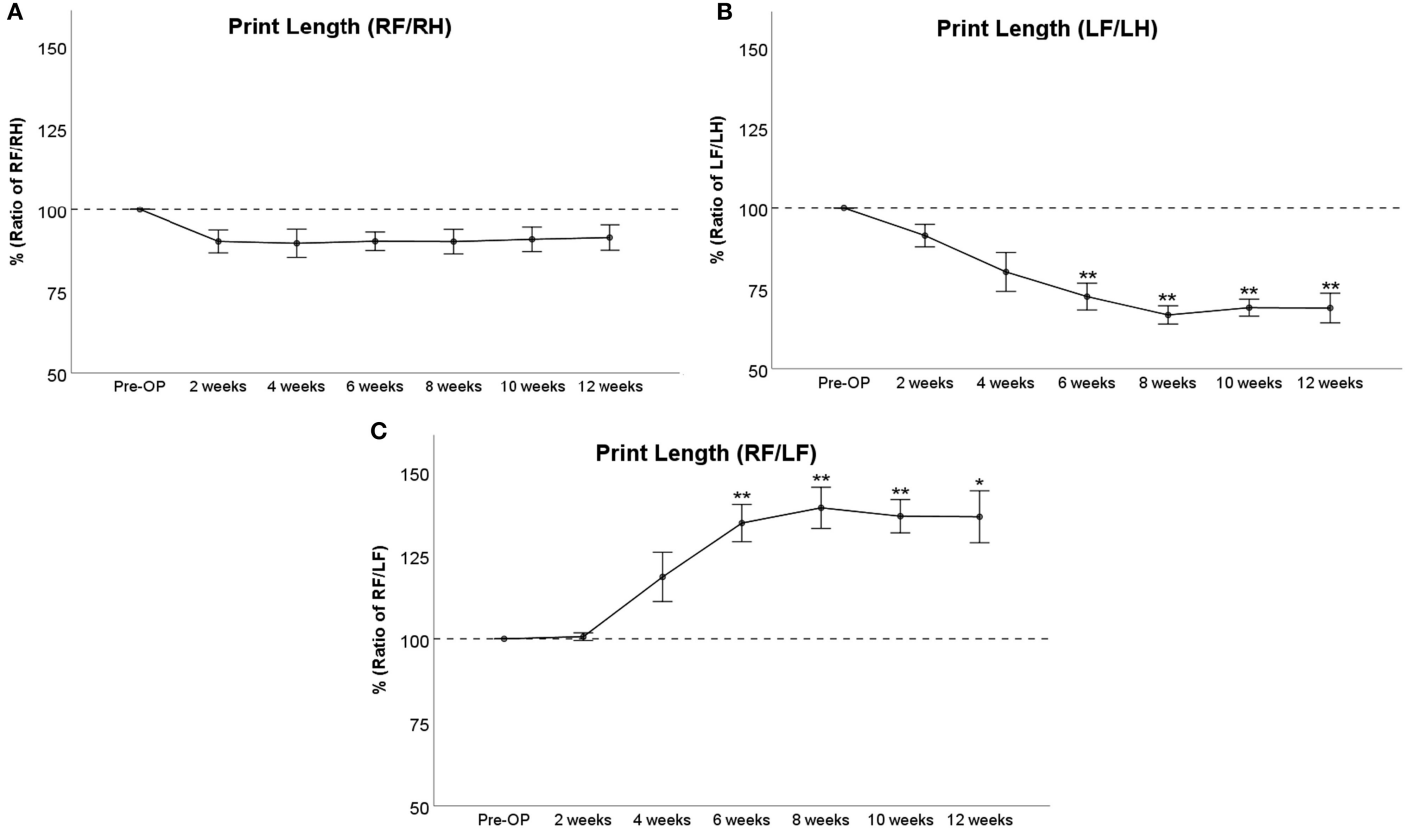
Course of Print Length following bilateral median nerve resection and unilateral autograft repair displayed as ratio of right front paw (RF)/right hind paw (RH) **(A)**, left front paw (LF)/left hind paw (LH) **(B)**, or RF/LF **(C)**, *p < 0.05 as compared to Pre-OP, **p < 0.01 as compared to Pre-OP.

RF/RH Print Length (Figure 3A) was decreased to around 90% of baseline from WPO2 until WPO12, but this was not statistically significant. LF/LH Print Length (Figure 3B) accounted for 91% and 80% of baseline at WPO2 and WPO4, respectively, which was not statistically significant. It was highly significantly (*p* < 0.01) reduced compared to baseline from WPO6 until the end of the observation period, reaching 72% at WPO6 and ranging between 69 and 67% at the following time points. RF/LF Print Length (Figure 3C) remained at 100% until WPO2, reached 120% of baseline at WPO4, and was significantly increased to 135% starting at WPO6 (*p* < 0.01). A further increment to 139% of baseline was observable at WPO8 (*p* < 0.01). The parameter remained elevated to 136% until the end of the observation period, which was significant compared to baseline at WPO10 (*p* < 0.01) and WPO12 (*p* < 0.05).

##### Print Width

**Figure 4:**
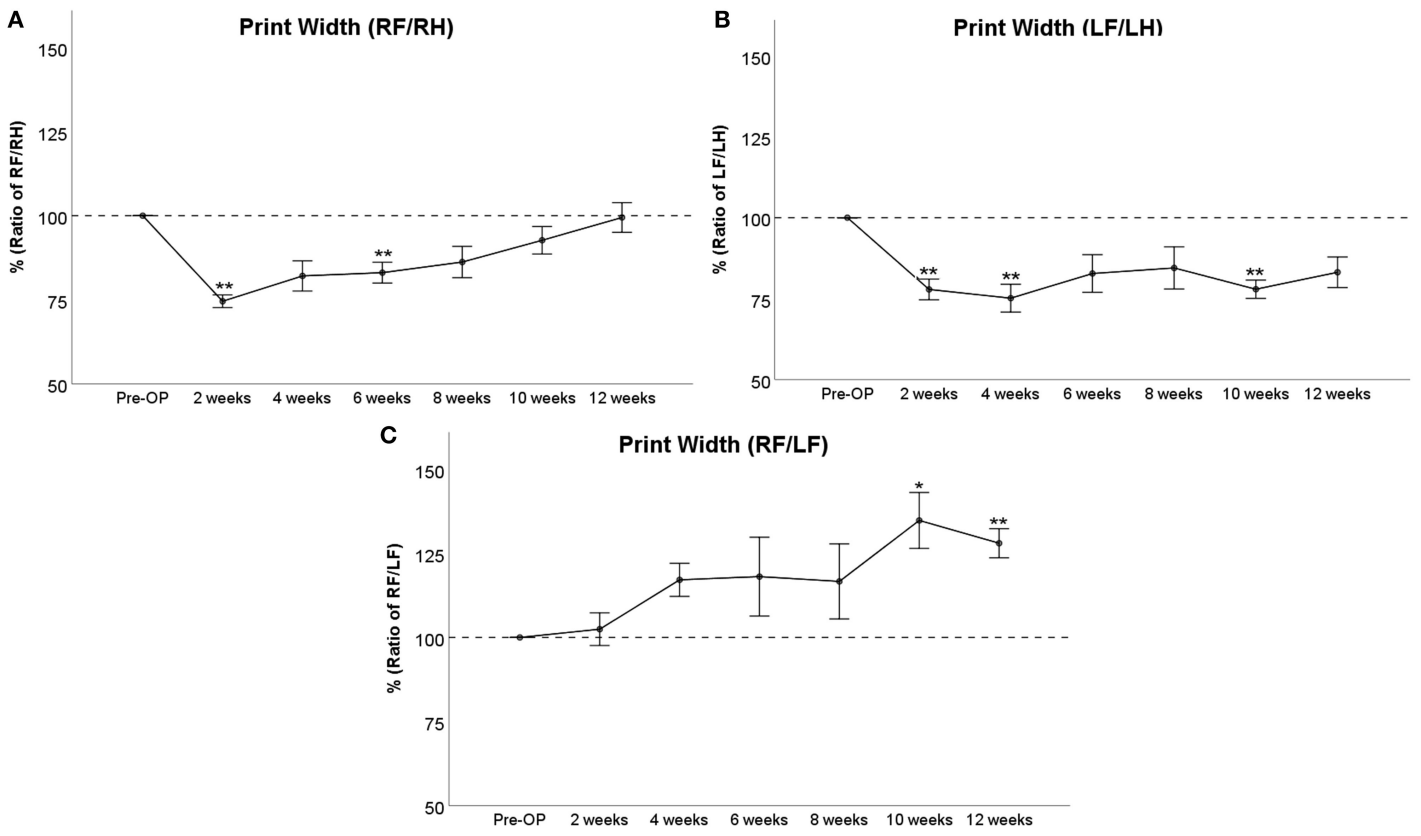
Evaluation of Print Width either of right front paw (RF) as compared to right hind paw (RH) **(A)**, of left front paw (LF) as compared to left hind paw (LH) **(B)**, or as a ratio of both front paws **(C)** following bilateral median nerve resection and unilateral autograft repair, *p < 0.05 as compared to Pre-OP, **p < 0.01 as compared to Pre-OP.

RF/RH Print Width (Figure 4A) was significantly decreased to 75% of baseline at WPO2 (*p* < 0.01). It increased to >80% at WPO4 and WPO6, which was still significantly altered from baseline in the case of the latter time point (*p* < 0.01). It was no longer significantly altered from baseline starting from WPO8 and recovered *ad integrum* at WPO12. LF/LH Print Width (Figure 4B) was highly significantly (*p* < 0.01) decreased to 78 and 75% of baseline at WPO2 and WPO4, respectively. It increased to 83 and 84% at WPO6 and WPO8, respectively. This was not significant compared to Pre-OP values. The parameter was highly significantly reduced to 78% at WPO10 (*p* < 0.01) before increasing to 83% at WPO12, which was not significantly reduced compared to baseline values. RF/LF Print Width (Figure 4C) was increased to 117–118% of baseline from WPO4 to WPO8, and a further increment to 135% of baseline was observable at WPO10, which was significant compared to Pre-OP values. While the parameter decreased to 128% at WPO12, it was highly significantly increased compared to baseline given the also decreased SEM (*p* < 0.01).

##### Base of Support

**Figure 5:**
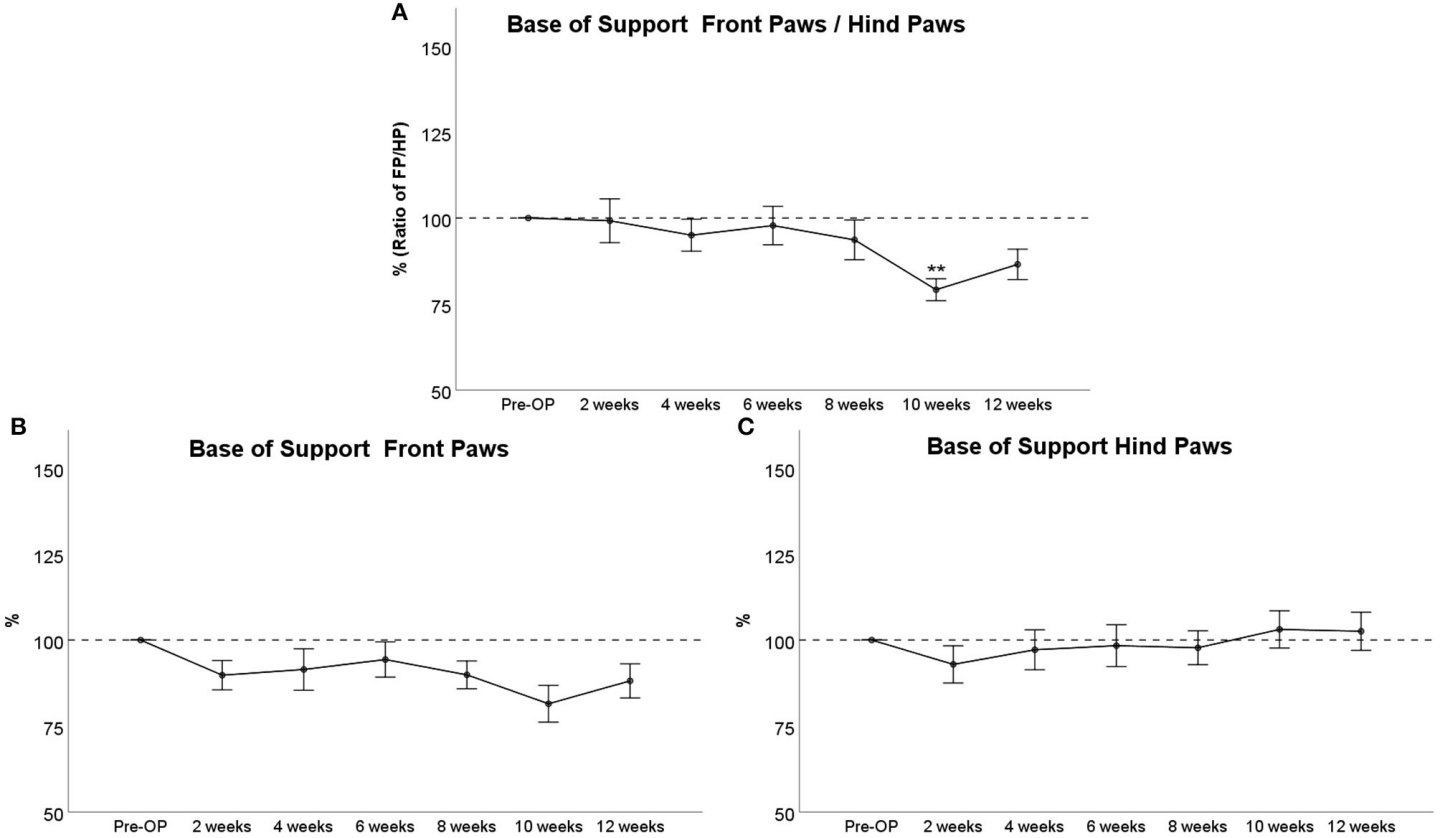
Course of Base of Support (BoS) prior to and following bilateral median nerve resection and unilateral autograft repair. The figures depict either the BoS Ratio between front paws and hind paws **(A)**, the course of front paw BoS **(B)**, or hind paw BoS **(C)**, **p < 0.01 as compared to Pre-OP.

The BoS Ratio between Front and Hind Paws (Figure 5A) decreased <10% following injury until WPO8. Noteworthy, there was a significant decrement to 80% at WPO10, which was highly significant compared to baseline (*p* < 0.01). Front paw/Hind paw (FP/HP) BoS increased again at WPO12 and was no longer significantly altered compared to baseline at this time point. BoS of the Front Paws (Figure 5C) was decreased about 10% following injury with a marked decrement at WPO10 in accordance with the aforementioned observations. There were no significant alterations from baseline values observable. Hind Paw BoS (Figure 5B) decreased to about 95% of baseline at WPO2, which was also the maximum decrement during the course of the observation period. Starting from WPO10, Hind Paw BoS reached higher values than at baseline and remained elevated to about 105% of Pre-OP values at WPO12. There were no significant differences to baseline measurements at any time point.

##### Stride Length

Stride Length (data not shown), by means of RF/RH, LF/LH, as well as RF/LF, remained constantly at 100% during the entire observation period.

#### Swing Speed

RF/RH Swing Speed (Supplementary Figure 1A) was decreased <10% compared to baseline values following surgery, but this was not significant. LF/LH Swing Speed (Supplementary Figure 1B) decreased to 94 and 91% at WPO2 and WPO4, respectively, which was not statistically significant. At WPO6, the parameter further decreased to 90% of baseline. This difference to baseline values was significant (*p* < 0.05). LF/LH Swing Speed remained significantly changed from Pre-OP at WPO8 (*p* < 0.05), WPO10 (*p* < 0.01), and WPO12 (*p* < 0.05) with values of 89, 87, and 90%, respectively. RF/LF Swing Speed (Supplementary Figure 1C) increased starting from WPO4. It was increased more than 10% compared to the left side from WPO6 onward, but this was not significant compared to Pre-OP.

#### Pain-Related Parameters of Gait

##### Mean Paw Print Intensity

RF/RH Mean Paw Print Intensity (Supplementary Figure 2A) was decreased to 90% of baseline at WPO2 (*p* = 0.052) and reached 99% of baseline at WPO12 again. There were no statistically significant differences observable compared to baseline. LF/LH Mean Paw Print Intensity (Supplementary Figure 2B) ranged between 96 and 103% of baseline until the end of the observation period. This was not significant compared to baseline at any time point. RF/LF Mean Paw Print Intensity (Supplementary Figure 2C) was decreased <5% following injury and reached around 100% of baseline at WPO10. There were also no significant differences observable compared to baseline.

##### Swing Time

RF/RH Swing Time (Supplementary Figure 3A) was increased around 10% but did not significantly change compared to baseline following surgery. While there were no significant alterations observable, it remained increased to 108% at WPO12. LF/LH Swing Time (Supplementary Figure 3B) increased to 109% of Pre-OP at WPO2 and reached 113 and 111% at WPO4 and WPO6, respectively. These differences were not statistically significant. The parameter increased to 113% at WPO8, which was statistically significant (*p* < 0.05) compared to baseline and to 115% at WPO10, which was highly statistically significant (*p* < 0.01). The value of 114% at WPO12 was not significantly altered from baseline. RF/LF Swing Time (Supplementary Figure 3C) started to decrease from WPO4 onward and was decreased about 10% compared to baseline from WPO6 until WPO12. There were no significant changes observable compared to baseline.

##### Duty Cycle

**Figure 6:**
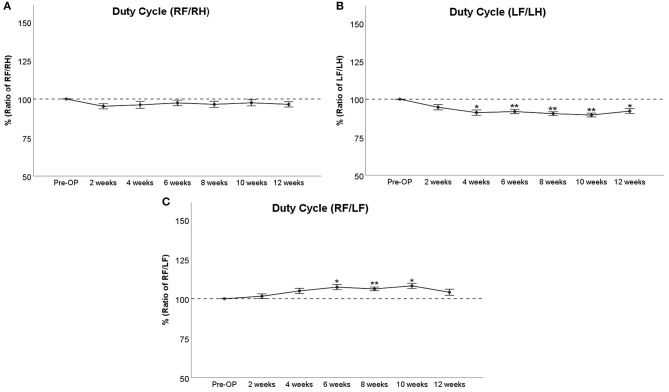
Assessment of Duty Cycle following median nerve resection and autograft repair. The panels depict the comparison of right front paw (RF) to right hind paw (RH) **(A)**, left front paw (LF) to left hind paw (LH) **(B)**, or RF to LF **(C)**, *p < 0.05 as compared to Pre-OP, **p < 0.01 as compared to Pre-OP.

RF/RH Duty Cycle (Figure 6A) was decreased <10% following injury until WPO10, which was not significant compared to baseline measurements. LF/LH Duty Cycle (Figure 6B) was reduced around 5% at WPO2, which was not significant compared to baseline. The parameter further decreased to 91% at WPO4 and ranged between 92 and 90% from WPO6 to WPO10. These values were significantly (*p* < 0.05) lower than Pre-OP measurements in case of WPO4 and highly significantly (*p* < 0.01) lower in the case of the latter two time points. LF/LH Duty Cycle slightly increased to 92% at WPO12, which was still significantly (*p* < 0.05) altered from baseline. RF/LF Duty Cycle (Figure 6C) of the RF started to increase at WPO2 and was significantly altered to around 107% of baseline at WPO6 and WPO10 (*p* < 0.05) as well as highly significantly altered from baseline at WPO8 (*p* < 0.01). The parameter decreased to 104% of baseline at WPO12. This was no longer significant compared to baseline.

#### Stand Index

**Figure 7:**
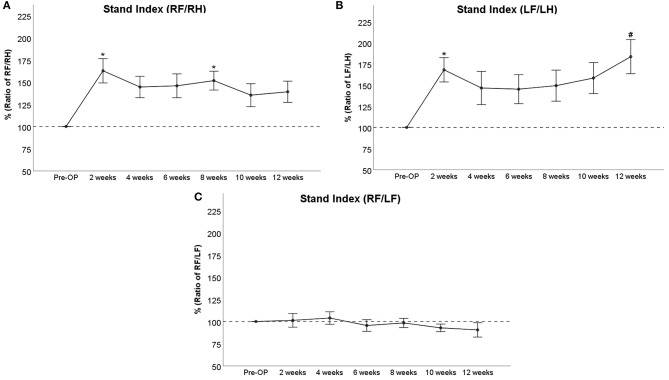
Stand Index as a ratio of right front paw (RF)/right hind paw (RH) **(A)**, ratio of left front paw (LF)/left hind paw (LH) **(B)**, or ratio of RF/LF **(C)**, *p < 0.05 as compared to Pre-OP, ^#^*p* = 0.053 as compared to Pre-OP.

RF/RH Stand Index (Figure 7A) was increased more than 1.5-fold compared to baseline at WPO2 (*p* < 0.05) and remained elevated between 145 and 150% until WPO8 (*p* < 0.05). The parameter decreased to around 140% of baseline at WPO10 and WPO12, which was not significantly altered compared to Pre-OP measurements. LF/LH Stand Index (Figure 7B) significantly (*p* < 0.05) increased to almost 168% at WPO2, followed by a decrement to 147% at WPO4. In the case of the latter time point, this was not significantly different from baseline values. LF/LH Stand Index ranged between 145 and 158% from WPO6 to WPO10, with no significant differences to Pre-OP. At WPO12, the parameter increased to 184% again, which was an almost significant (*p* = 0.053) difference to baseline measurements. RF/LF Stand Index (Figure 7C) was slightly elevated to <105% of baseline following injury but started to decrease between WPO4 and WPO6. It reached 90% of baseline at WPO12. There were no significant differences observable compared to baseline.

#### Coordination-Related Parameters of Gait

**Figure 8:**
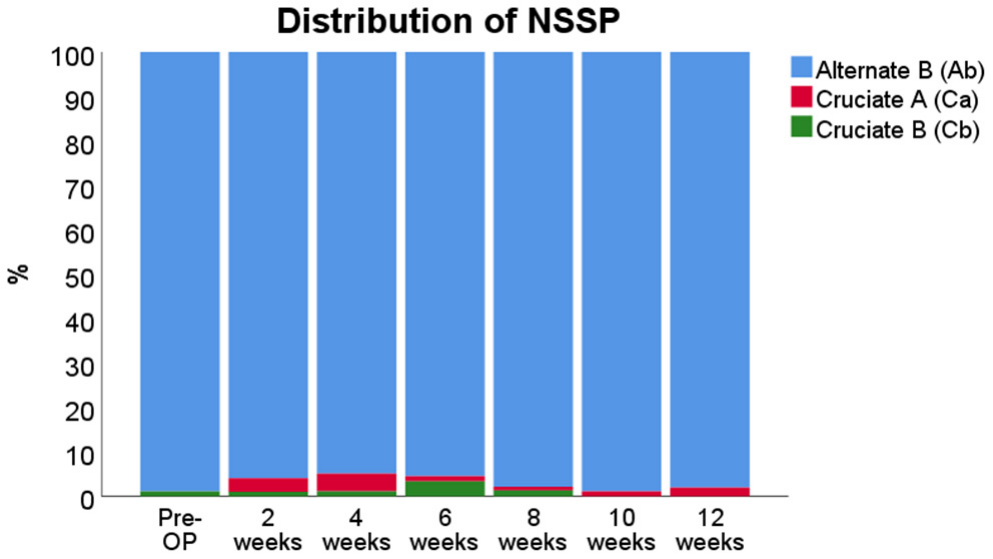
Distribution of Normal Step Sequence Patterns (NSSPs) before and after median nerve neurotmesis and autograft repair.

Regarding the coordination-related parameters, evaluation of the RI revealed no relevant changes and the RI remained constant between 99.6 and 100% over the entire course of the observation period (data not shown). All six Phase Dispersions/Couplings showed no alterations from baseline during the course of the observation period throughout WPO12 (data not shown). In terms of the NSSPs (Figure 8), the alternate pattern b (Ab) was used by all rats between 99 and 100% prior to median nerve resection and autograft repair. Starting from WPO2, rats increased their use of the two cruciate patterns a (Ca) and b (Cb). The Ca pattern was used in about 4–5% of cases until WPO4 and the Cb in the remaining 1–2% of non-Ab patterns. This shifted at WPO6, when rats used the Cb pattern in 4–5% of cases, accompanied by 1–2% of Ca. Starting from WPO8, use of the Cruciate patterns ceased, with only 1% of each respective pattern belonging to this category at WPO10. Use of the two Cruciate patterns was almost negligible at WPO10 but increased again at WP012 when use of the Ca pattern was accountable for all 4% of non-Ab patterns.

#### External Paw Rotation

**Figure 9:**
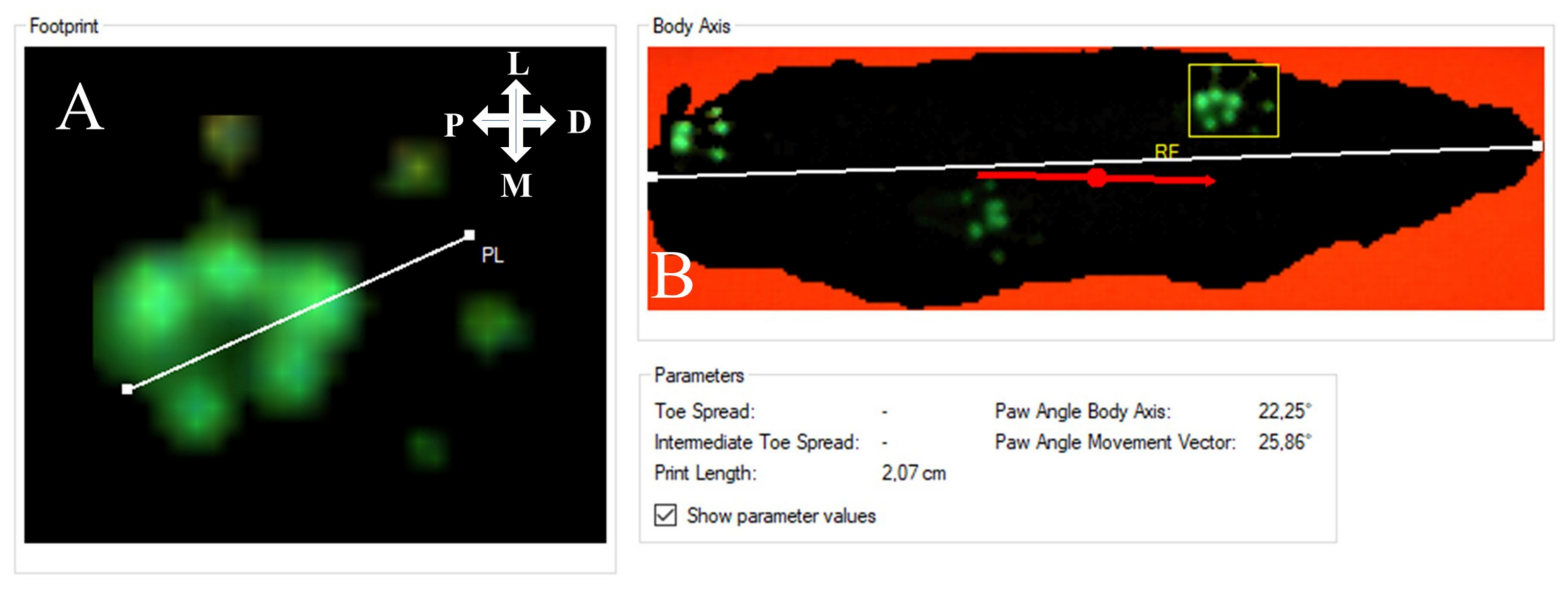
Evaluation of the degree of external paw rotation by measuring the Paw Angle Body Axis. This is the angle between the orientation of the front paw **(A)** and the animal's body axis **(B)**. Note that both axes can be individually drawn by the experimenter analyzing the CatWalk (CW) data. **(A)** The white line defines the orientation of the paw. **(B)** The white line defines the animal's body axis. The red line defines the animal's body movement vector, which is calculated by linear regression of the animal's center of gravity in the recorded frame and in the three frames preceding it. The white cross indicates the orientation of the respective paw in relation to the rat's body axis; P, proximal; D, distal; M, medial; L, lateral.

**Figure 10:**
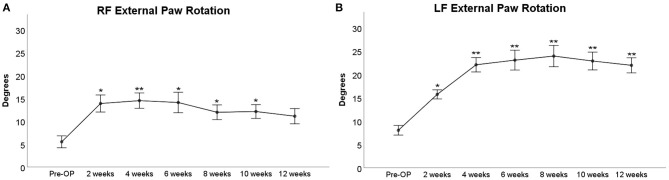
External paw rotation between the right front **(A)** or left front paw **(B)** and the rat's body axis prior to and following median nerve neurotmesis and autograft repair, *p < 0.05 as compared to Pre-OP, **p < 0.01 as compared to Pre-OP.

Since we observed a marked external rotation of both front paws following median nerve injury in the operated animals, we were curious to evaluate if these findings could on the one hand be related to loss of function of the median nerve and functional recovery following nerve repair on the other hand. The degree of external paw rotation is assessable by determining the PABA, which measures the angle between the animal's body axis (defined as the line between the tip of the nose and the base of the tail) and LF or RF (Figure 9). This analysis revealed distinct courses of this parameter for the right (Figure 10A) and left side (Figure 10B), respectively. While the Pre-OP PABA was 5° in case of RF and 8° in case of LF, both PABAs were significantly increased to 14° (RF) and 16° (LF), respectively, at WPO2 (*p* < 0.05). In case of LF, the PABA increased to 22° at WPO4, which was highly statistically significant in comparison to baseline measurements (*p* < 0.01). It remained highly significantly (*p* < 0.01) elevated between 22° and 23° for the rest of the observation period. PABA of RF showed an increment to 14.5° at WPO4, which was only a slight increase compared to WPO2, but highly significant compared to Pre-OP values (*p* < 0.01). It was still elevated to 14° at WPO6 but decreased to 12° at WPO8 and WPO10. This was statistically significant in comparison to Pre-OP measurements for all three aforementioned time points (*p* < 0.05). A further decrement was observable at WPO12 when PABA of RF was 11°, which was no longer significant compared to baseline values. Linear regression analysis of the right FDS muscle weight and external paw rotation revealed a significant correlation between both parameters (*p* = 0.048, *R*^2^ = 0.406).

### Reflex-Based Grasping

#### Participation in the Testing Procedure

**Figure 11:**
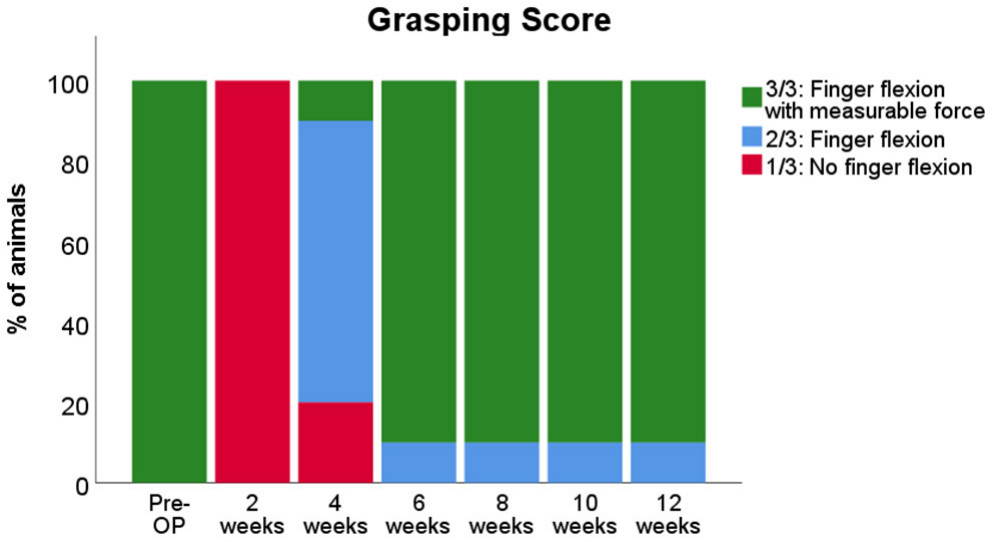
Evaluation of the grasping ability prior to and following median nerve resection and autograft repair in rats. Note that although all rats recovered the ability of finger flexion with measurable force by 8 weeks post-operatively (WPO8), some animals displayed reduced motivation to participate in the reflex-based grasping test at various time points, indicated by the percentage of animals that showed finger flexion without measurable force from WPO6 throughout WPO12.

All 10 animals that underwent biweekly evaluation of grasping strength (Figure 11) regained the ability to flex their fingers (2/3 on the rating scale) at WPO6 the latest. Earliest evidence of motor recovery—the ability to grasp the bar—was shown by one animal at WPO4. At WPO8, all animals were able to pull the bar of the testing apparatus with measurable grasping force (3/3). However, several animals displayed reduced motivation to participate in the testing procedure over the course of the observation period. While they showed the ability to pull the bar with measurable force (3/3) While they showed the ability to pull the bar with measurable force (3/3) at one measurement time point, e.g. WPO6, they were reluctant to pull it the following week, resulting in a score of 2/3 based on Stossel's scale. In total, 40% of the animals demonstrated this behavior at least once over the course of the observation period. Therefore, these animals had to be excluded, and only six animals were eligible to be included for further statistical analysis of the measured mean and maximum grasping strength by means of RMANOVA.

#### Grasping Strength

**Figure 12:**
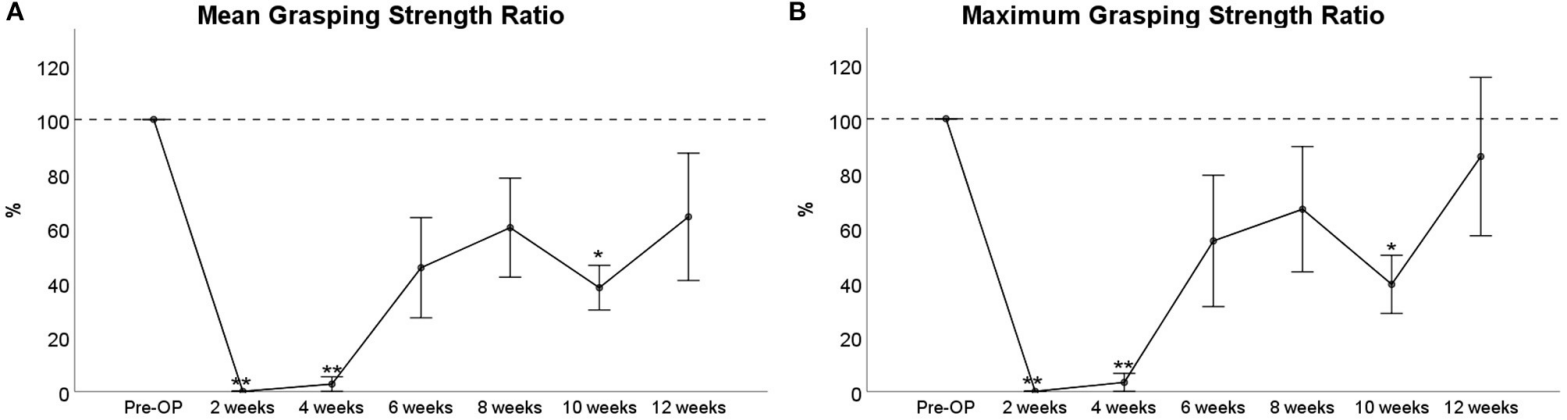
Assessment of mean **(A)** and maximum **(B)** grasping strength following 7-mm median nerve resection and autograft repair. Note the reduced number of animals (*n* = 6) that could be included for statistical testing by means of repeated measures ANOVA (RMANOVA). This was due to reduced motivation to participate in the testing procedure. *p < 0.05 as compared to Pre-OP, **p < 0.01 as compared to Pre-OP.

The mean grasping strength (Figure 12A) was reduced to 0% of Pre-OP values at WPO2 and 3% at WPO4, respectively, which was highly significant compared to baseline values (*p* < 0.01). It increased to 35% at WPO6, which was no longer significant compared to Pre-OP values and showed a further increase to 60% at WPO8. Noteworthy, mean grasping strength decreased to 38% again at WPO10. The difference to baseline values was statistically significant at this time point (*p* < 0.05). At WPO12, values increased to 64% again and were no longer significantly altered from baseline.

Analysis of the maximum grasping strength (Figure 12B) revealed similar results. While the parameter was significantly reduced to 0% and 3% of baseline values at WPO2 and WPO4, respectively (*p* < 0.01), maximum grasping strength increased to 55% at WPO6 and further to 67% at WPO8, which was no longer statistically significant compared to baseline values. In accordance with the course of mean grasping strength, a decrement to 39% of baseline was observable at WPO10 (*p* < 0.01). The maximum grasping strength increased to 86% of Pre-OP values at WPO12 again, which was not significant compared to Pre-OP measurements.

### Electrophysiological Measurements

Electrophysiological evaluations (*n* = 9) of CMAP amplitude and CMAP latency revealed a mean CMAP amplitude of 10 mV (SEM = 0.21) and a mean CMAP latency of 1.5 (SEM = 0.44) ms at WPO12 following 7-mm median nerve resection and autograft repair.

### Linear Regression Analysis of Electrophysiology and Functional Data

Linear regression analyses (*n* = 9) between the electrophysiological measurements and each of the assessed CW parameters at WPO12 (Supplementary Table 1) revealed a statistically significant correlation between the CMAP latency and the BoS FP/HP ratio (*p* = 0.045, *R*^2^ = 0.459) and a statistically significant correlation between CMAP amplitude and RF External Paw Rotation (*p* = 0.020, *R*^2^ = 0.088). Correlations close to statistical significance were revealed between CMAP latency and RF/LF Print Area (*p* = 0.059, *R*^2^ = 0.420), CMAP amplitude and FP BoS (*p* = 0.054, *R*^2^ = 0.549), and CMAP latency and BoS HP (*p* = 0.054, *R*^2^ = 0.434). Linear regression analyses (*n* = 6) featuring electrophysiological parameters and mean grasping strength as well as maximum grasping strength (Supplementary Table 1) revealed no significant results.

### Correlation Analysis Between CatWalk Data and Grip Strength Measurements

Correlation analyses by means of calculation of Pearson's correlation coefficient between CW data on the one hand and mean grasping strength as well as maximum grasping strength on the other hand (Supplementary Table 2) revealed a highly significant correlation between Print Area RF/RH and mean/maximum grasping strength (*p* < 0.01). There was also a highly significant correlation between RF/RH Print Length and mean as well as maximum grasping strength (*p* < 0.01). RF/RH Print Width showed a high correlation with maximum grasping strength only (*p* < 0.01). BoS of the Hind Paws correlated highly significantly with both strength measures as well (*p* < 0.01). RF/RH Swing Speed (*p* < 0.01) and the two pain-related parameters RF/RH Duty Cycle (*p* < 0.05 and *p* < 0.01, respectively) and RF/RH Swing Time (*p* < 0.05) also correlated significantly with mean grasping strength and maximum grasping strength. The degree of RF External paw rotation as assessed *via* PABA calculation showed a highly significant correlation (*p* < 0.01) with mean grasping strength as well as a significant correlation with maximum grasping strength (*p* < 0.05).

### Correlation Analysis Between CatWalk Parameters

Correlation analysis by calculation of Pearson's correlation coefficients between CW data (Supplementary Table 3) was performed to draw conclusions regarding possible interrelations of the various assessed parameters. The analysis revealed a highly significant degree of correlation between several parameters (Supplementary Table 4). A high degree of correlation was observable (1) between the general parameters of gait, e.g., Print Area, Print Length, and Print Width as well as (2) between the pain-related parameters of gait such as Mean Paw Print Intensity, Duty Cycle, and Stand Index. There was also a highly significant degree of correlation between RF/RH Print Area and RF/LF Duty Cycle as well as RF/RH Print Area and RF/RH Stand Index. We also observed such correlations between RF/LF Print Area and RF/RH Duty Cycle. RF/RH Print Length correlated highly significantly with RF/LF Mean Paw Print Intensity, RF/LF Duty Cycle, RF/RH Stand Index, and RF/LF Stand Index. There was a highly significant correlation between RF/RH Print Width, RF/LF Duty Cycle, and External Paw Rotation of RF on the one hand and between RF/LF Print Width and RF/RH Duty Cycle on the other hand. All three BoS measures correlated with various general as well as several pain-related parameters of gait. RF/RH Swing Speed was highly significantly correlated with RF/LF Mean Paw Print Intensity, RF/LF Duty Cycle, RF/LF Stand Index, and RF/RH Swing Time. RF/LF Swing Speed displayed a highly significant degree of correlation with RF/RH Duty Cycle, RF/LF Duty Cycle, RF/RH Swing Time, and RF/LF Swing Time.

### Multivariate Analysis of Outcome Measures by Principal Component Analysis

**Figure 13:**
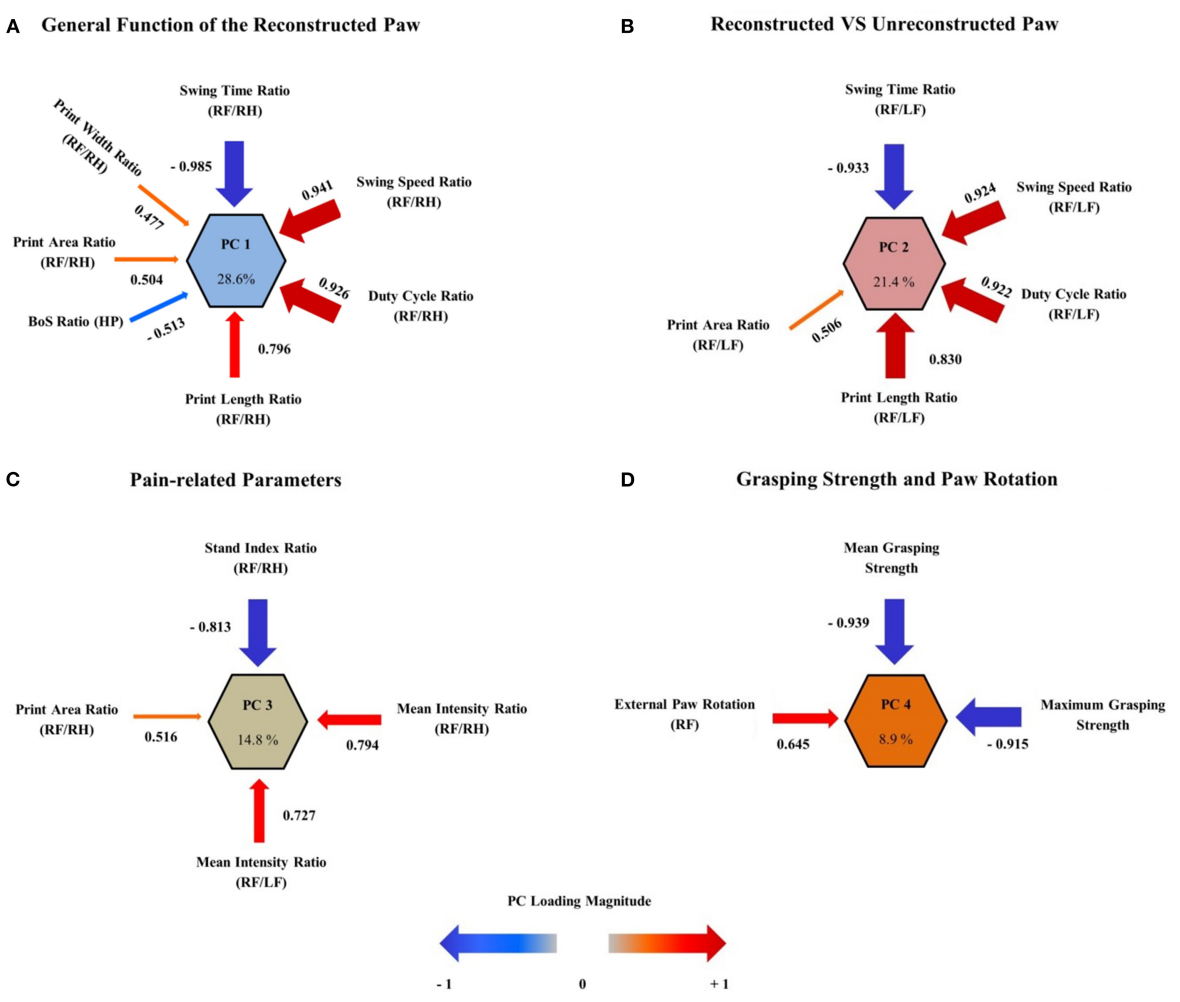
Multivariate analysis of outcome parameters. By using principal component analysis (PCA), four principal component clusters (PCs) were extracted, which accounted for 73.6% of the variance in observed outcome after median nerve resection and autograft repair. The correlation (also referred to as “loadings”) of each outcome metric on the PC is represented as arrows, with the arrow size indicating the loading magnitude and the color indicating the valence of the loading. PC1 **(A)** represents the largest variance (28.6%) and relates to the general function of the reconstructed right front paw. PC2 **(B)** accounts for 21.4% of variance and represents the ratio of functionality between the reconstructed right and unreconstructed left front paw. The third primary component cluster, PC3 **(C)** contains four pain-related parameters of gait, reflecting the influence of pain perception on functional outcome. This component accounts for 14.8% of variance. PC4 **(D)** represents 8.9% of variance and reflects the muscle function of the long finger flexors (maximum and mean grasping strength) as well as of the flexor carpi radialis muscle (external paw rotation). Note that external paw rotation has a direction of loading opposed to the direction of loading of grasping strength, indicating the decrement of external paw rotation with increasing muscle reinnervation. The size of the arrows corresponds to the loading magnitude, while the color corresponds to the direction of loading.

To evaluate the roles of CW data and grasping strength as outcome metrics after median nerve resection and autograft repair, we performed multivariate analysis of variables by means of PCA. The purpose of PCA is to identify a limited number of principal components that represent the overall variance of all variables included in the analysis. PCA was performed on all variables included in Supplementary Table 3 and revealed four principal component clusters (PC1–4) which accounted for 73.6% of the variance in outcome in our study (Figures 13A–D). **PC1** (Figure 13A), the largest cluster, accounted for 28.6% of variance with the following variables loading onto it: (1) RF/RH Swing Time (−0.985), (2) RF/RH Swing Speed (0.941), (3) RF/RH Duty Cycle (0.926), (4) RF/RH Print Length (0.796), (5) HP BoS (−0.513), (6) RF/RH Print Area (0.504), and (7) RF/RH Print Width (0.477). **PC2** (Figure 13B) represented 21.4% of variance of outcome metrics and (1) RF/LF Swing Time (−0.933), (2) RF/LF Swing Speed (0.924), (3) RF/LF Duty Cycle (0.922), (4) RF/LF Print Length (0.830), and (5) RF/LF Print Area (0.506) loaded onto it. **PC3** (Figure 13C) and **PC4** (Figure 13D) accounted for 14.8 and 8.9% of variance, respectively. While the four variables (1) RF/RH Stand Index (−0.813), (2) RF/RH Mean Paw Print Intensity (0.794), (3) RF/RH Print Area (0.516), and (4) RF/LF Mean Paw Print Intensity (0.727) loaded onto PC3, (1) RF External Paw Rotation (0.645), (2) Mean Grasping Strength (−0.939), and (3) Maximum Grasping Strength (−0.915) loaded onto PC4.

## Discussion

With this work, we provide the first comprehensive analysis of gait behavior *via* the CW method in rats following 7-mm median nerve neurotmesis with and without autograft repair. The results of our correlation analyses between CW data and two other commonly used assessment methods in the rat median nerve model—grasping strength measurements and electrophysiological evaluations—have also not been reported before. In the following section, we will discuss the integral parameters one by one.

### CatWalk Gait Analysis

#### General Parameters of Gait

In regard to the general parameters of gait, distinct changes were observed. Print Area and Print Width were strongly decreased upon injury, and both recovered *ad integrum* in case of the RF/RH ratio at WPO12. Both Print Area and Print Width of RF were also significantly increased compared to LF, indicating the effects of autograft repair vs. a non-reconstructed defect. These findings are in line with those of other authors (Chen et al., 2012a,b) who have also reported strongly decreased Print Area and Print Width following 2-mm median nerve resection and concomitant nerve ligation. Noteworthy, these authors did only investigate changes of gait until 28 days post operation (DPO28) without reconstruction of the median nerve; therefore, no functional recovery was observable. Interestingly, in the study by Chen et al. Print Area and Print Width showed a marked increase following administration of baclofen, an analgesic drug that ameliorates symptoms of painful neuropathy (Terrence et al., 1985), indicating a possible correlation between the aforementioned parameters and neuropathic pain. The courses of LF/LH Print Area and LF/LH Print Width indicate a significant loss of paw contact area until WPO4, followed by an increase in the following 4 to 6 weeks. Another significant decrement in Print Width and Print Area of the unreconstructed paw became observable at WPO10 and WPO12, respectively. As demonstrated by Bao et al. (2018), median nerve injury results in a stronger activation of the corresponding primary motor area and sensory cortex at WPO2, contrasted by a markedly decreased activation at 2 months following injury. According to these findings, we conclude that the same phenomenon is underlying the observations made in our study. Therefore, in our opinion, the initial decrement in Print Area and Print Width could be related to the development of neuropathic pain in the context of increased central sensory representation of both affected paws. This also fits the observation that RF/RH parameters started to increase at WPO4 already, a time point which corresponds to the reinnervation of the paw by regenerating axons. The second decrement at WPO10 and WPO12, respectively, corresponds well to the aforementioned observed decreased representation of the unreconstructed LF in the sensorimotor cortex, resulting in diminished use of the respective paw. Interestingly, the authors of a study investigating the time course of neuroma formation following forelimb amputation in a rodent model reported the strongest histological evidence of neuroma formation between DPO60 and DPO90 (Oliveira et al., 2018). The level of amputation in these authors' study corresponds very well to the level of median nerve transection in our study. Additionally, we did not suture the proximal nerve stump of the left median nerve to any nerve or muscle, further increasing the likelihood of neuroma formation. Since it has been previously mentioned by other authors and our group that the perception of pain on the general parameters of gait should be assumed as an influencing factor, we hypothesize that this “late decrement” could very well also be related to neuroma pain. Although we did not perform histological assessments of the left median nerve to verify neuroma formation, intraoperative pictures of the left proximal median nerve stump (Supplementary Figure 4) support this assumption.

In our study, Print Length of RF was decreased less strongly than Print Area and Print Width of the same paw following injury, which can be explained by the fact that the length of the paw print is defined by the distance between the heel and the most distal toe. As median nerve injury did not result in absent detection of all toes, Print Length was only slightly reduced. The observed increase of RF/LF Print Length to more than 135% is therefore in line with an increased weight support on the second and third digits, which in turn increases RF Print Length in comparison to the chronically denervated contralateral front paw. Noteworthy, the course of LF/LH Print Length showed a marked decrease following WPO2, contrasting the relatively slight decrement of the right side. In our opinion, this observed non-use of the LF toes during gait can also be explained by the combination of increased cortical representation of the affected paw and the development of neuropathic pain. The observed minimal values starting from WPO8 might also be a consequence of accentuated neuroma development and reduced use of the respective paw due to decreased cortical representation.

RF/RH, LF/LH, and RF/LF Stride Length remained unchanged from baseline, which can be explained by the fact that this parameter is the product of Swing Speed and Swing Time, which both were altered around 5–10% at WPO2 compared to baseline. This kind of counter-regulatory mechanism has been reported in other rat studies of peripheral nerve injury (PNI) (Deumens et al., 2007; Bozkurt et al., 2011; Lemke et al., 2018) and is thought to allow the rat to maintain superimposed placement of ipsilateral paws during locomotion to secure safe footing and balance (Hruska et al., 1979; Heinzel et al., 2020b). However, it remains to be elucidated which of the two parameters, Swing Time or Swing Speed, was initially more affected by median nerve neurotmesis and subsequently counterbalanced by an alteration of the other one. Based on findings in rodent models of hind limb PNI, e.g., sciatic nerve and femoral nerve injury, the movement of the limb during the swing phase is mainly mediated by nerves that innervate the muscles of the proximal limb, e.g., the sciatic nerve and femoral nerve innervate the extensor muscles and flexor muscles of the proximal hind limb, respectively (Jackson, 1936; Irintchev, 2011). Innervation of the proximal forelimb muscles is facilitated not by the median nerve but other branches of the brachial plexus such as the suprascapular nerve, musculocutaneous nerve, and radial nerve (Bertelli et al., 1992, 1995). The median nerve supplies muscular fibers to the flexor muscles of the forearm and digits and sensory fibers to the medial aspect of the paw and medial digits (Jackson, 1936). Based on these anatomical considerations, we hypothesize that changes of Swing Time and Swing Speed are rather related to alterations of sensory function than loss of function of muscles, which play a key role during the swing phase of the respective forelimb. Given the fact that Swing Time was categorized as a pain-related parameter of gait (Deumens et al., 2007) and alterations in Swing Speed are more likely related to loss of motor function (Heinzel et al., 2020b), we suggest that Swing Time is the parameter mainly affected by median nerve neurotmesis, which is than counterbalanced by a decrement of Swing Speed. Noteworthy, both LF/LH Swing Time and Swing Speed were significantly altered from baseline from WPO8 to WPO10 and WPO6 to WPO12, respectively. As these changes were highly significant at WPO10, this further supports our hypothesis of underlying pain due to manifested neuroma formation as the main reason for the increase in Swing Time at this time point.

The course of BoS following median nerve injury and repair showed no significant alterations with the exception of WPO10, when FP BoS decreased, leading to a significant decrease of the FP/HP BoS ratio. Whereas an increased BoS was reported to account for an unstable gait in rats with spinal cord injury (Hamers et al., 2001; Joosten et al., 2004; Datto et al., 2016), it was shown to strongly decrease in rats with sciatic nerve neurotmesis (Deumens et al., 2007) and to show slight fluctuations in rats with femoral nerve neurotmesis (Heinzel et al., 2020b). In light of the only slight alterations observable in our model, we hypothesize that these changes might also be related to the way the BoS is calculated by the CW software. Since the BoS is defined as the distance between the center of the paw prints, BoS is strongly affected by a general decrease in Print Width, as observed after median nerve neurotmesis. Following autograft repair and subsequent reinnervation, RF Print Width increased as the medial digits become detected again by the CW software, resulting in an increased BoS. In our opinion, the observed alterations in BoS are less likely related to impaired locomotor or sensory function in general but are a consequence of altered paw prints due to median nerve injury.

#### Pain-Related Parameters of Gait

In regard to the pain-related parameters of gait, Mean Paw Print Intensity did not show any significant changes compared to baseline over the entire observation period in our model. This contrasts immunohistochemical (Lue et al., 2002; Tsai et al., 2004, 2009; Yeh et al., 2008; Lin et al., 2012) and behavioral studies (Chen et al., 2012a,b; Shaikh et al., 2016) of neuropathic pain following median nerve neurotmesis. Chen et al. (2012a) have reported a 20% decrease in Mean Paw Print Intensity at DPO3 following 2-mm median nerve resection and concomitant ligation, which recovered to >90% of baseline at DPO7. The same authors have reported significant alterations of neuropeptide Y and c-Fos expression (Chen et al., 2012b), which are additional indicators of neuropathic pain, following the aforementioned injury pattern. Expression of both neuropeptide Y and c-Fos was detectable in the cuneate nucleus (Yeh et al., 2008) as early as 5 days after median nerve neurotmesis, peaked at WPO4, and was reduced to baseline levels at WPO16 (Lue et al., 2002; Tsai et al., 2004). Interestingly, gait analysis did not show maximal alterations from baseline at WPO4 but WPO2 instead, indicating that immunohistochemical findings might follow a different course than behavioral changes following PNI. This is underpinned by the findings of (Chen et al., 2012a) who have reported a maximum decrement of Mean Paw Print Intensity at DPO3. Although these authors performed median nerve ligation, which is known to induce painful neuropathy (Kim and Chung, 1992; Kim et al., 1997) in addition to nerve transection, they found the same spatiotemporal expression patterns of neuropeptide Y and c-Fos as reported in the aforementioned studies. Besides neuropathic symptoms caused by injury of the median nerve, the adjacent nerves, e.g., the ulnar and radial nerve should—in our opinion—also be considered in this context. Collateral sprouting of sensory nerve fibers from adjacent territories, e.g., the saphenous nerve in case of sciatic nerve injuries, has been reported to cause early hyperalgesia and mechanical allodynia as soon as 3 days following injury followed by late hyperalgesia when regenerating axons of the “original” nerve reinnervate the respective skin area (Cobianchi et al., 2013, 2014). Since cutaneous innervation of the palmar front paw skin is provided by both the median and ulnar nerve (Jackson, 1936; Barton et al., 2017), it remains to be elucidated if a similar mechanism occurs following median nerve injury. Therefore, we advise for upcoming studies of neuropathic pain in rats with median nerve neurotmesis to correlate results of functional assessment methods with those of immunohistochemical evaluations of both the median nerve and ulnar nerve. Noteworthy, we did not detect any behavioral signs of the aforementioned late hyperalgesia as has been described by (Cobianchi et al., 2013, 2014).

Regarding the other pain-related parameters in our study, RF/RH Duty Cycle was slightly reduced following median nerve neurotmesis and reconstruction, whereas LF/LH Duty Cycle was significantly decreased from WPO4 until WPO12, indicating a strong effect of unreconstructed median nerve neurotmesis on weight support on the affected paw. We hypothesize that the significant alterations of RF/LF Duty Cycle at WPO6–WPO10 can be explained by sensory reinnervation of RF by the regenerating median nerve. We conclude that the resulting amelioration of pain symptoms became most evident at WPO8 when the RF/LF ratio was highly significantly altered from baseline, further supporting the aforementioned hypothesis regarding the effects of most prominent neuroma formation at WPO8. Although other signs of hyperalgesia, e.g., a strong decrement of Mean Paw Print Intensity, were absent in our study, our findings are limited by the relative late time point when we began to evaluate gait behavior at WPO2 and the lack of additional evaluation methods for pain-related symptoms. Therefore, future studies should feature an early-on assessment of gait behavior or comparable assessment methods such as Von Frey testing or an analgesymeter (Bertelli et al., 2004; Casal et al., 2020) in order to detect signs of early hyperalgesia and mechanical allodynia. Given the fact that sensory perturbations, i.e., hypersensitivity to hot, cold, and mechanical stimuli, have been reported to occur in both front paws following sham surgery of the right median nerve in rats, central mechanisms of pain mediation are also an interesting area of research in our opinion (Shaikh et al., 2016). From our point of view, these findings also raise concern regarding the use of sham-operated animals as an inter-animal control group.

The parameter Stand Index, which measures the speed with which the paw loses contact with the floor (Batka et al., 2014; Boix et al., 2018) has been shown to increase in rats with neuropathic pain (Kameda et al., 2017). RF/RH Stand Index was also markedly increased in our study. This might be related to painful neuropathy but could also be linked to the overall reduced Print Area following nerve injury, resulting in a reduction of the time required for paw liftoff. Interestingly, RF/RH Stand Index showed an accentuated decrement at WPO10 and WPO12, indicating slower paw liftoff in comparison to previous time points. Again, it remains to be elucidated in further studies whether this is linked to amelioration of pain symptoms, an overall increase in Print Area, or a combination of both. The course of LF/LH Stand Index can be interpreted as an argument for the influence of pain on the parameter, as it displayed a marked increase at WPO10 and WPO12, reaching almost a significant difference to baseline values at the latter time point. In accordance with our aforementioned hypothesis regarding neuroma formation at WPO8, we argue that neuroma-related pain is probably the underlying reason for the observable increase in LF/LH Stand Index from WPO8 onward. As the right median nerve was reconstructed with an autograft in our study, this increase is absent in case of RF/RH Stand Index, resulting in an overall decrease of RF/LF Stand Index. In our opinion, these considerations further support the parameter's feasibility as a marker for pain influence on gait in rats with peripheral nerve injuries.

#### Coordination-Related Parameters of Gait

The coordination-related parameters RI and all six pairs of Phase Dispersions remained unchanged from baseline following median nerve injury. In terms of the RI, this is in accordance with previous studies, which have shown that this parameter is affected by central (Koopmans et al., 2005; Crowley et al., 2018) instead of peripheral nervous injuries (Bozkurt et al., 2011; Heinzel et al., 2020b), underlining the fact that the center for control of inter-limb coordination is located within the central nervous system (Alford et al., 2003; Kyriakou et al., 2015). Phase Dispersions/Couplings were altered from baseline, both in rat studies of sciatic (Bozkurt et al., 2011) and femoral nerve resection (Heinzel et al., 2020b), as well as central nervous injuries (Hetze et al., 2012) and neurodegenerative diseases (Boix et al., 2018). Changes in these coordination-related parameters have been linked to neuroplasticity following nerve injury (Navarro et al., 2007). We therefore hypothesize that median nerve injury results in less accentuated changes of proprioception of the affected paw, given the small area of the limb innervated by the median nerve compared to both the femoral or sciatic nerve (Jackson, 1936). Changes in the distribution of NSSP, specific sequences of paw placements during locomotion (Cheng et al., 1997; Deumens et al., 2014), have been observed in rats after both sciatic nerve and femoral resection and are thought to correspond to altered proprioception of the respective limb (Heinzel et al., 2020b). To maintain balanced gait, rats tend to let the affected limb be preceded by the most distant paw, e.g., LF is preceding RH in case of resection of the right sciatic nerve (Deumens et al., 2007). A similar phenomenon was observable in our model, although to a lesser extent. Rats with bilateral median nerve injury showed increased use of the two cruciate patterns, with a preference for the Ca pattern that relieves LF (unreconstructed side) until WPO4. This was followed by a preference for the Cb pattern that relieves RF at DPO6. Interestingly, the latter finding corresponded well to an increase in Print Width of RF/LF. We therefore hypothesize that altered proprioception of the two partially denervated front limbs was mainly responsible for changes in NSSP distribution until WPO4. Corresponding to sensory reinnervation of RF, the distribution of NSSP changed again as rats were trying to relieve the now differently perceived RF. Some use of the Ca pattern still remained until WPO12, which corresponds well to the fact that LF remained chronically denervated with consequent chronically altered proprioception in our model.

### Methods for Assessment of Motor Reinnervation

#### Grasping Strength

Besides CW evaluation of sensory reinnervation after median nerve injury by means of parameters like Print Area, identification of parameters that allow for simultaneous evaluation of muscular reinnervation would further increase comprehensiveness of the method from our and other authors' points of view (Kappos et al., 2017). As the degree of functional recovery is the most important criterion to determine the effect of any neuroregenerative treatment approach (de Medinaceli et al., 1982), this would further contribute to increase clinical translation of observations and findings made in the rat median nerve model. For this purpose, we performed correlation analysis of functional data, aiming to pinpoint potential parameters that significantly correlate with well-established assessment methods of motor reinnervation in the median nerve model. Evaluation of motor recovery in the rat median nerve model (Galtrey and Fawcett, 2007; Ronchi et al., 2009, 2019) is most commonly performed by means of the grasping test (Bertelli and Mira, 1995; Papalia et al., 2003; Nichols et al., 2005; Casal et al., 2020). However, we experienced some major limitations of this method in our study. Most importantly, the grasping test does not allow for standardization of the speed with which the animals are lifted after grasping the bar that therefore depends on the individual investigator (Tos et al., 2009). Recent efforts to circumvent this problem by stimulated grasping strength measurements while the animal is in anesthesia are a valuable refinement (Hanwright et al., 2019). However, data acquisition is further complicated by this approach, which in our opinion is also concerning regarding the need for weekly anesthesia of the animals. Second, Lewis rats show a lower rate of automutilation following PNI (Carr et al., 1992) and might therefore be preferred over other rat strains such as Sprague–Dawley or Fischer rats. However, they display a reduced motivation to participate in repetitive testing procedures, leading to unreproducible results of the grasping test (Stossel et al., 2017). This limitation was impressively confirmed in our experiments, in which 40% of animals had to be excluded from RMANOVA analysis of grasping strength assessments. As shown by our as well as other authors' data (Daeschler et al., 2018b; Marchesini et al., 2018; Hanwright et al., 2019), a high degree of inter-animal variation is observable, complicating statistical analysis of the assessed data. In regard to our study, it is noteworthy that grasping strength was significantly altered from baseline at WPO2 and WPO4, statistically indifferent from baseline at WPO6 and WPO8, but markedly decreased at WPO10 before increasing to baseline levels again at WPO12. As this was paralleled by a marked increase of RF/LF Print Area at WPO10, we hypothesize that the observed decrement in grasping strength at WPO10 was related to changes in sensory qualities of the respective paw, e.g., alteration of proprioception or the perception of pain. It has been emphasized by other authors that reflex-based grasping is a sensory–motor response circuit, involving both sensory afferents and motor efferents (Navarro, 2016; Stossel et al., 2017). Last but not least, the way how the grasping test is performed might induce stress in the animals, a cofounding factor for most behavioral assessments (Sestakova et al., 2013; Rabl et al., 2016). Given the aforementioned limitations and pitfalls of the grasping test to evaluate functional recovery, we postulate the need for complementary outcome measures of motor reinnervation following median nerve injury. One promising new parameter, external paw rotation, is presented in the following section.

#### External Paw Rotation

We hereby propose external paw rotation, which is assessable *via* CW gait analysis as a new parameter to evaluate muscular reinnervation in rats with median nerve injury. Paw rotation has already been reported as a sensitive marker for functional recovery in rats with sciatic nerve injury (Varejao et al., 2003, 2004). In our study, this parameter was significantly changed from baseline following median nerve neurotmesis, reached baseline values in case of the reconstructed RF at WPO12, and remained highly significantly altered from Pre-OP values in case of the chronically denervated LF. Additionally, the parameter showed a significant (*p* < 0.01) correlation with both mean and maximum grasping strength as well as wet weight of the FDS muscle. We hypothesize that increased external paw rotation results from a muscular imbalance (Millesi and Tsolakidis, 2005; Millesi, 2006) between the flexor carpi ulnaris muscle, innervated by the ulnar nerve, and the flexor carpi radialis muscle (FCR), innervated by the median nerve (Jackson, 1936; Bertelli et al., 1995). Following denervation of the FCR after median nerve neurotmesis in our study, the paw was externally rotated due to muscular traction of the flexor carpi ulnaris muscle. This muscular imbalance became more accentuated with ongoing denervation time of the FCR, mirrored by the increase of external paw rotation at WPO4. As the FCR became reinnervated by regenerating axons following autograft repair, external paw rotation of RF decreased again but showed a further increase in case of the chronically denervated LF. These findings are also in line with the significant negative correlation between FDS weight and external paw rotation by means of linear regression analysis. As the muscle weights of the FDS and FCR increased due to reinnervation, external paw rotation decreased. Noteworthy, Casal et al. (2018, 2020), who evaluated gait behavior in rats with median nerve injury *via* an “ink and paper” method, have reported a negative correlation between radial deviation, i.e., internal paw rotation and FCR muscle weight, linking higher weight of the FCR to increased external rotation of the paw. Although this seems to contradict our findings, it must be noted that the aforementioned authors have explicitly mentioned that wrist kinematics in rats are most likely not attributable to one muscle in isolation and might also be influenced by other muscles innervated by the median nerve such as the thenar and lumbrical muscles as well as the pronator teres muscle. Noteworthy, studies investigating the role of these muscles during locomotion are sparse and additional research is necessary to improve understanding of their interaction during gait. We also would like to explicitly mention the conflicting reports regarding innervation of the forelimb muscles in the rat (Barton et al., 2017). While some authors have reported that the long finger flexors are innervated by the median nerve alone (Bertelli et al., 1995; Tos et al., 2009), Greene has described a mixed innervation pattern comprising both the median and ulnar nerves (Jackson, 1936). The same discrepancies apply to the pronator teres muscle, which different authors have reported to be innervated either by both the median and musculocutaneous nerves (Bertelli et al., 1995) or exclusively by the musculocutaneous nerve (Jackson, 1936). Innervation of the FCR has been reported to stem exclusively from either the median nerve (Bertelli et al., 1995) or musculocutaneous nerve (Jackson, 1936). In light of these contradictory reports, we recommend further anatomical studies to be conducted to investigate in detail the aforementioned innervation patterns and their variants. In our opinion, this would help shed light on the neuroanatomy underlying a myriad of experimental studies.

### Correlation of Outcome Measures

The observed degree of correlation between functional assessment methods (grasping strength and CW data) and electrophysiological measurements as well as wet muscle weight revealed a significant positive correlation between RF/LF Stand Index and CMAP amplitude. This indicates that faster paw liftoff occurs in rats with improved electrophysiological properties of the FCS, contrasting the observation of a general down-trend of this parameter, e.g., slower paw liftoff during the course of the observation period. In our opinion, this phenomenon could be linked to a faster liftoff movement, e.g., finger flexion during liftoff mediated by the FCS. Therefore, reinnervation of the FCS results in improved finger flexion, an important movement sequence during paw liftoff (Wang et al., 2008). This hypothesis also underpins the influence of both motor and sensory reinnervation on functional recovery following PNI, e.g., an overall reduction of the Stand Index due to sensory reinnervation and ameliorated perception of pain is contrasted by an increase of the Stand Index due to motor reinnervation of the FCS. Regarding the correlation between the different parameters of gait assessable *via* CW, our results confirmed the customary grouping of parameters into different groups as listed in Table 1. Whereas, the general parameters of gait were significantly intercorrelated on the one hand, the same was true for the pain-related parameters of the other hand. Interestingly, Print Length, Print Width, and Print Area were also correlated with several pain-related parameters, emphasizing that changes of the paw print dimensions correspond to both alterations in sensory perception in general and the sensation of pain in the respective paw (Deumens et al., 2007; Heinzel et al., 2020b). Stand Index did also correlate significantly with both general and pain-related parameters of gait, further supporting our hypothesis that this parameter is affected by recovery of motor and sensory function as well as by the perception of pain. This is also emphasized by the positive correlation between RF/RH Stand Index and RF/RH Duty Cycle, which indicates a faster paw liftoff while stand time on the respective paw is also increasing. In our opinion, this further supports our hypothesis that the parameter Stand Index also measures motor nerve regeneration, e.g., faster paw liftoff due to muscular reinnervation. In case the parameter would mainly be influenced by the perception of pain, there should be a significantly negative correlation between Stand Index and Duty Cycle.

Linear regression analysis of electrophysiological and functional evaluations revealed a significant correlation between CMAP amplitude and RF/LF Stand Index as discussed before. There was also a significant correlation between CMAP latency and FP/HP BoS, which in our opinion has to be interpreted with great caution. One could hypothesize that CMAP latency might serve as a surrogate marker for the conductance velocity of sensory fibers as well (Bhatt et al., 2017); therefore, an increase in latency could correspond to a slower conductance velocity of sensory afferents. As slower conductance of sensory afferents might correspond to slower recovery of sensory perception and Print Area in consequence, this might serve as an explanation for the observed degree of correlation, given our consideration of how BoS is affected by Print Area. A strong trend toward correlation between CMAP and RF/LF Print Area further supports this hypothesis. Furthermore, we provided proof for a significant degree of correlation between Mean and Maximum grasping strength, respectively, and the recovery, e.g., RF/RH ratio, of various CW parameters such as Print Area, Print Length, Swing Speed, and Duty Cycle. There was also a significant degree of correlation between RF External Paw Rotation and grasping strength measurements, further underpinning our aforementioned hypothesis that the latter parameter allows for evaluation of motor reinnervation in rats with median nerve injury.

### Principal Component Clusters of Outcome Measures

To gain a better understanding of how different outcome metrics fit within the overall functional outcome after bilateral median nerve resection and unilateral autograft repair, we performed PCA of CW data and grasping strength measurements of the reconstructed RF. Although PCA is rarely applied in preclinical research in general, it has been proven a feasible method for integration of multiple outcome metrics in rat models of cervical spinal cord contusion injury (Irvine et al., 2014). In the aforementioned study, PCA has been used to integrate outcome metrics from several evaluation methods [histology; Irvine, Beatties, and Bresnahan (IBB) Forelimb Recovery Scale; open field testing; paw placement; and CW]. We used it to integrate outcome metrics of CW testing and the grasping test only, revealing four PCs, which related to different aspects of functional recovery following median nerve injury. While PC1 represented the general recovery of paw function indicated by the loading of the general parameters of gait, PC2 indicated the relation of functional differences between the reconstructed right and unreconstructed LF. PC3 represented the perception of pain indicated by the loading of several pain-related parameters. Noteworthy, mean grasping strength and maximum grasping strength loaded on PC4 as well as external paw rotation of the RF, which indicated that this PC represents the motor reinnervation following median nerve injury and repair. These findings emphasize that the conventional CW parameters measure different features of functional recovery than the grasping test. This is in line with the findings of Irvine et al. (2014), who have emphasized that the CW most likely captures other functional deficits than assessment tools that measure function of the forelimb, paws, and/or digits in segregation. This is underpinned by the consideration that quadrupedal gait involves four limbs and multiple muscles instead of the one limb and two muscles involved in finger flexion. Our results also underscore that the proposed parameter of external paw rotation has a significant degree of construct validity, given its loading of >0.6 on PC4 together with both mean and maximum grasping strength.

### Limitations

Despite that we were able to show the CW method's feasibility to assess functional recovery in rats after median nerve resection and autograft repair, several limitations need to be addressed. First of all, the CW's character as a complementary method must be emphasized. It has been emphasized by us as well as other authors that its solitary use cannot replace complex batteries of behavioral and functional analysis tools such as locomotor recovery scales, open field locomotion, etc. (Irvine et al., 2014; Heinzel et al., 2020a). This is especially relevant in cases of central nervous system injuries, such as spinal cord contusion. Such injuries evoke functional deficits that are more complex than those after isolated peripheral nerve injuries. As our study lacked the aforementioned wide variety of functional outcome assessments, subsequent experiments should be performed to evaluate the correlation of CW data and other tools for assessment of functional recovery. Second, in rat studies of spinal cord contusion injury, the CW method's results did not correlate with histological metrics of nerve regeneration, one of the most commonly used outcome criteria in animal studies of nerve injury (Wood et al., 2011; Irvine et al., 2014). Since our study did not elucidate the relationship between histological and functional metrics of nerve regeneration, we suggest that this limitation should also be tackled in subsequent experiments.

## Conclusion

Evaluation of functional recovery by means of CW automated gait analysis is a widely established method in rodent models of PNI (Deumens et al., 2014; Kappos et al., 2017). However, it has never been used to study motor and sensory reinnervation in a rat model of median nerve resection and autograft repair. With this work, we were able to show that CW gait analysis is a feasible tool to evaluate functional recovery in rats with median nerve injury. Our work also highlights the differences in gait following median nerve resection with and without autograft repair. Given the complexity of quadrupedal locomotion in comparison to isolated flexion of the digits, the complementary role of the CW device for comprehensive evaluation of functional recovery in the rat median nerve model is further emphasized. Besides the CW's feasibility as a complementary assessment tool, we were also able to identify several parameters, Print Area, Duty Cycle, and Swing Time, which correlated well with other assessment methods such as grasping strength measurements and electrophysiology. Therefore, the CW device allows for comprehensive evaluation of functional deficits and recovery that do not become evident when the grasping test alone is used. Additionally, external paw rotation measures an outcome closely related to grasping strength while avoiding the aforementioned test's disadvantages and characteristics of a forced, non-voluntary behavior. Our results also indicate the potential of simultaneous evaluation of both motor and sensory recovery when evaluation of Print Area is combined with a measurement for muscle reinnervation such as the degree of external paw rotation. We are convinced that this work contributes to improve understanding of functional deficits and nerve recovery following forelimb nerve injury. These new insights will help researchers to improve experimental therapies and innovative approaches to enhance nerve regeneration or restore functionality by means of prosthesis following nerve injury.

## Data Availability Statement

The raw data supporting the conclusions of this article will be made available by the authors, without undue reservation.

## Ethics Statement

The animal study was reviewed and approved by City Government of Vienna Municipal Department 58.

## Author Contributions

JH conceptualized the work, performed the experimental surgeries, performed the gait analysis, classified parts of the CW data, contributed to all evaluations, analyzed the data, and wrote the manuscript. VO performed the gait analysis and classified the acquired CW data. CK assisted in experimental surgeries. NS performed the electrophysiological measurements. GL and HF performed the grasping tests. JK and CP critically revised the manuscript. JG and DH conceptualized the work, oversaw the experiments, and critically revised the manuscript. All authors read and approved the submitted final version of the manuscript.

## Conflict of Interest

The authors declare that the research was conducted in the absence of any commercial or financial relationships that could be construed as a potential conflict of interest.

## Abbreviations

CW: CatWalk
DPO: day(s) post-operatively
FCR: flexor carpi radialis muscle
FCU: flexor carpi ulnaris muscle
WPO: week(s) post-operatively
FP: front paw(s)
HP: hind paw(s)
RF: right front limb
LF: left front limb
LH: left hind limb
RH: right hind limb
PABA: paw angle body axis
PCA: principal component analysis
PC: principal component cluster
RMANOVA: repeated measures ANOVA
SEM: standard error of the mean.
