# Supplementary material for: Evaluation of Functional Recovery in Rats After Median Nerve Resection and Autograft Repair Using Computerized Gait Analysis
Source: Front Neurosci. 2021 Jan 21;14:593545. doi: 10.3389/fnins.2020.593545 (PMC7859340; doi:10.3389/fnins.2020.593545)
Supplement: Supplementary file 1 [file Table_1.DOCX]

**Supplementary Table 1** – Linear regression analysis of functional evaluation methods (CatWalk gait analysis and grasping strength measurements) and electrophysiological evaluations. Significant p-values (<0.05) are marked in bold. Close to significant p-values (0.05>p<0.06) are highlighted in grey.

| CatWalk parameter | CMAP amplitude | CMAP latency |
| --- | --- | --- |
| Print Area (RF/RH) | p= 0.283 (R^2^ = 0.162) | p= 0.877 (R^2^ = 0.004) |
| Print Area (RF/LF) | p= 0.425 (R^2^ = 0.093) | p= 0.059 (R^2^ = 0.420) |
| Print Length (RF/RH) | p= 0.691 (R^2^ = 0.024) | p= 0.640 (R^2^ = 0.033) |
| Print Length (RF/LF) | p= 0.903 (R^2^ = 0.002) | p= 0.102 (R^2^ = 0.336) |
| Print Width (RF/RH) | p= 0.604 (R^2^ = 0.041) | p= 0.137 (R^2^ = 0.287) |
| Print Width (RF/LF) | p= 0.119 (R^2^ = 0.310) | p= 0.517 (R^2^ = 0.062) |
| BoS Front Paws | p= 0.054 (R^2^ = 0.549) | p= 0.950 (R^2^ = 0.001) |
| BoS Hind Paws | p= 0.220 (R^2^ = 0.206) | p= 0.054 (R^2^ = 0.434) |
| BoS Front / Hind Paws | p= 0.669 (R^2^ = 0.028) | **p= 0.045** (R^2^ = 0.459) |
| Swing Speed (RF/RH) | p= 0.575 (R^2^ = 0.047) | p= 0.860 (R^2^ = 0.005) |
| Swing Speed (RF/LF) | p= 0.791 (R^2^ = 0.001) | p= 0.544 (R^2^ = 0.055) |
| Mean Intensity (RF/RH) | p= 0.921 (R^2^ = 0.001) | p= 0.454 (R^2^ = 0.083) |
| Mean Intensity (RF/LF) | p= 0.995 (R^2^ = 0.000) | p= 0.973 (R^2^ = 0.000) |
| Duty Cycle (RF/RH) | p= 0.744 (R^2^ = 0.016) | p= 0.678 (R^2^ = 0.026) |
| Duty Cycle (RF/LF) | p= 0.933 (R^2^ = 0.001) | p= 0.329 (R^2^ = 0.136) |
| Swing Time (RF/RH) | p= 0.471 (R^2^ = 0.077) | p= 0.924 (R^2^ = 0.001) |
| Swing Time (RF/LF) | p= 0.895 (R^2^ = 0.003) | p= 0.342 (R^2^ = 0.129) |
| Stand Index (RF/RH) | p= 0.183 (R^2^ = 0.237) | p= 0.107 (R^2^ = 0.328) |
| Stand Index (RF/LF) | **p= 0.020** (R^2^ = 0.563) | p= 0.993 (R^2^ = 0.000) |
| RF External Paw Rotation | p= 0.438 (R^2^ = 0.088) | p= 0.849 (R^2^ = 0.006) |
| Mean grasping strength | p= 0.694 (R^2^ = 0.059) | p= 0.530 (R^2^ = 0.143) |
| Maximum grasping strength | p= 0.782 (R^2^ = 0.030) | p= 0.581 (R^2^ = 0.151) |
