# Supplementary material for: Evaluation of Functional Recovery in Rats After Median Nerve Resection and Autograft Repair Using Computerized Gait Analysis
Source: Front Neurosci. 2021 Jan 21;14:593545. doi: 10.3389/fnins.2020.593545 (PMC7859340; doi:10.3389/fnins.2020.593545)
Supplement: Supplementary file 2 [file Table_2.DOCX]

**Supplementary Table 2** – Correlation analysis between CatWalk data and grasping strength measurements. All Pearson correlation coefficients were calculated over 42 individual data points. pcc: Pearson correlation coefficient. Significant p-values (<0.05) are marked in bold.

| CatWalk parameter | Mean grasping strength | Maximum grasping strength |
| --- | --- | --- |
| Print Area (RF/RH) | **p= 0.001** (pcc = 0.572) | **p= 0.001** (pcc = 0.563) |
| Print Area (RF/LF) | p= 0.145 (pcc = - 0.229) | p= 0.311 (pcc = - 0.160) |
| Print Length (RF/RH) | **p= 0.001** (pcc = 0.531) | **p= 0.001** (pcc = 0.495) |
| Print Length (RF/LF) | p= 0.895 (pcc = 0.021) | p= 0.727 (pcc = 0.056) |
| Print Width (RF/RH) | p= 0.060 (pcc = 0.293) | **p= 0.043** (pcc = 0.314) |
| Print Width (RF/LF) | p= 0.170 (pcc = - 0.216) | p= 0.339 (pcc = - 0.151) |
| BoS Front Paws | p= 0.173 (pcc = - 0.214) | p= 0.140 (pcc = - 0.232) |
| BoS Hind Paws | **p= 0.005** (pcc = - 0.426) | **p= 0.007** (pcc = - 0.408) |
| BoS Front / Hind Paws | p= 0.294 (pcc = 0.166) | p= 0.363 (pcc = 0.144) |
| Swing Speed (RF/RH) | **p= 0.002** (pcc = 0.458) | **p= 0.002** (pcc = 0.458) |
| Swing Speed (RF/LF) | p= 0.899 (pcc = 0.020) | p= 0.771 (pcc = 0.046) |
| Mean Intensity (RF/RH) | p= 0.410 (pcc = 0.131) | p= 0.384 (pcc = 0.138) |
| Mean Intensity (RF/LF) | p= 0.305 (pcc = - 0.162) | p= 0.219 (pcc = - 0.194) |
| Duty Cycle (RF/RH) | **p= 0.014** (pcc = 0.376) | **p= 0.001** (pcc = 0.396) |
| Duty Cycle (RF/LF) | p= 0.214 (pcc = - 0.196) | p= 0.318 (pcc = - 0.158) |
| Swing Time (RF/RH) | **p= 0.018** (pcc = - 0.362) | **p= 0.021** (pcc = - 0.355) |
| Swing Time (RF/LF) | p= 0.544 (pcc = 0.096) | p= 0.691 (pcc = 0.063) |
| Stand Index (RF/RH) | p= 0.071 (pcc = - 0.282) | p= 0.129 (pcc = - 0.238) |
| Stand Index (RF/LF) | p= 0.184 (pcc = 0.209) | p= 0.173 (pcc = 0.214) |
| RF External Paw Rotation | **p= 0.010** (pcc = - 0.391) | **p= 0.046** (pcc = - 0.310) |
