# Supplementary figures and images for: Evaluation of Functional Recovery in Rats After Median Nerve Resection and Autograft Repair Using Computerized Gait Analysis
Source: Front Neurosci. 2021 Jan 21;14:593545. doi: 10.3389/fnins.2020.593545 (PMC7859340; doi:10.3389/fnins.2020.593545)

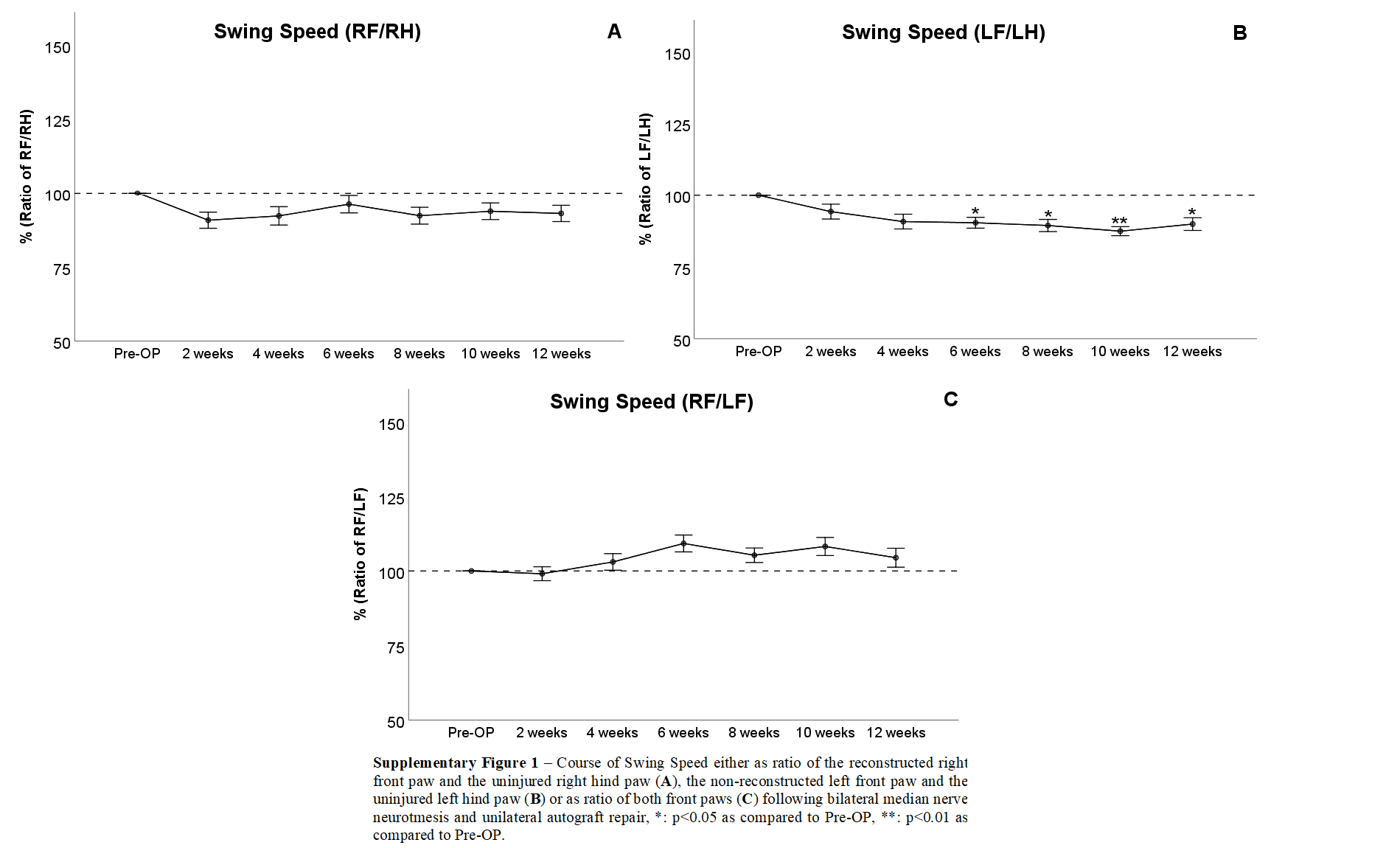

Supplement: Supplementary file 5 [file Image_1.TIFF]

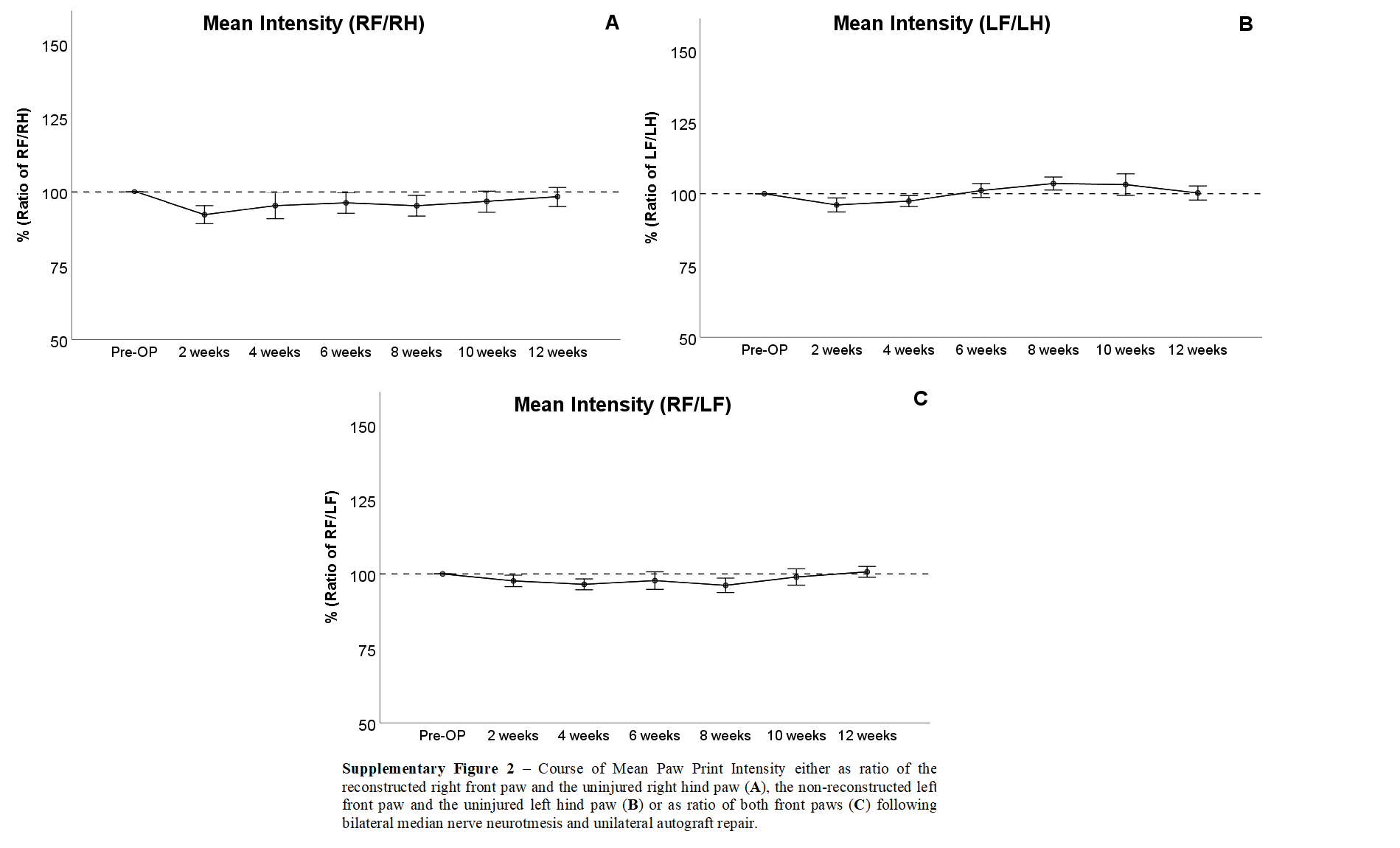

Supplement: Supplementary file 6 [file Image_2.PNG]

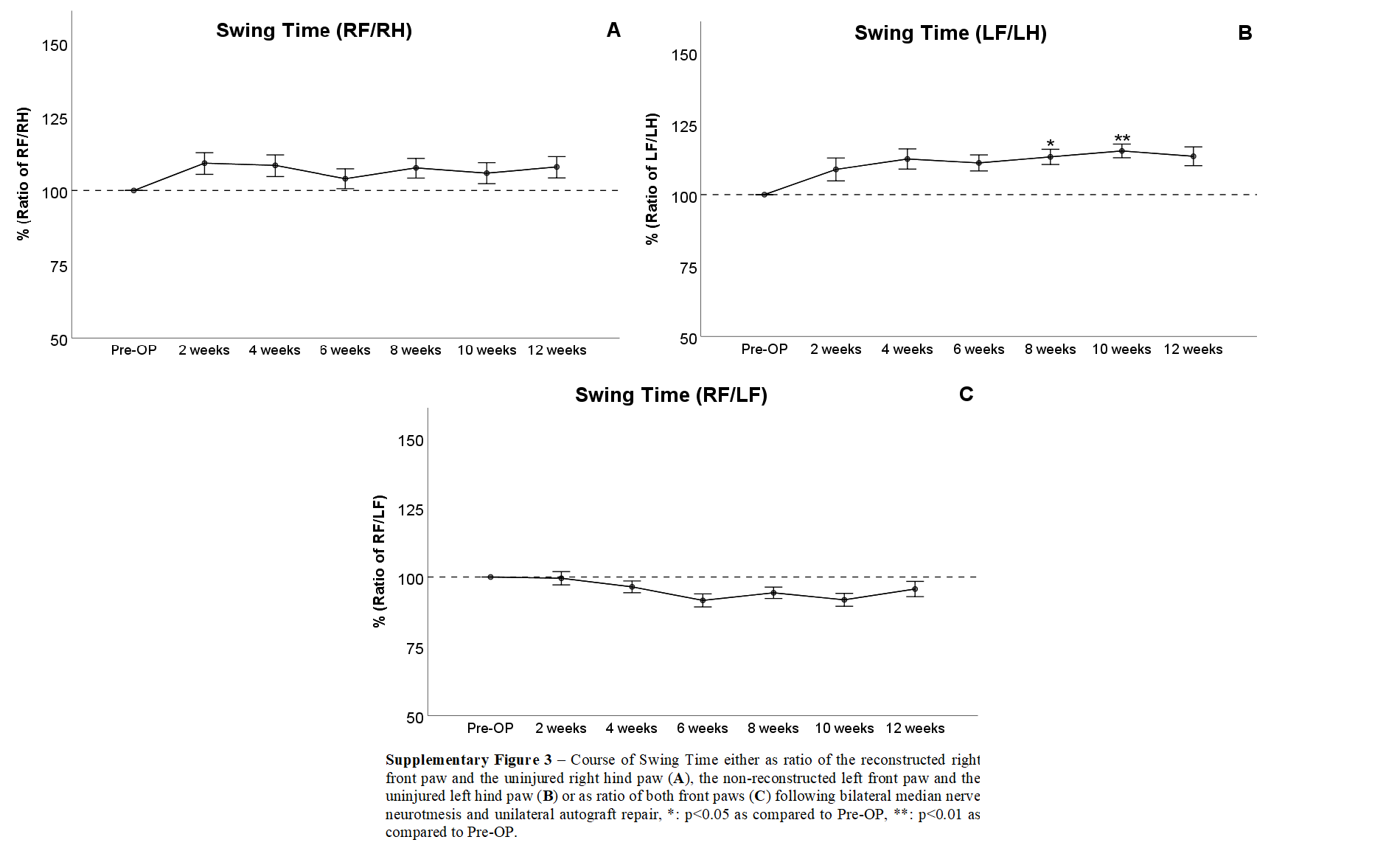

Supplement: Supplementary file 7 [file Image_3.TIFF]

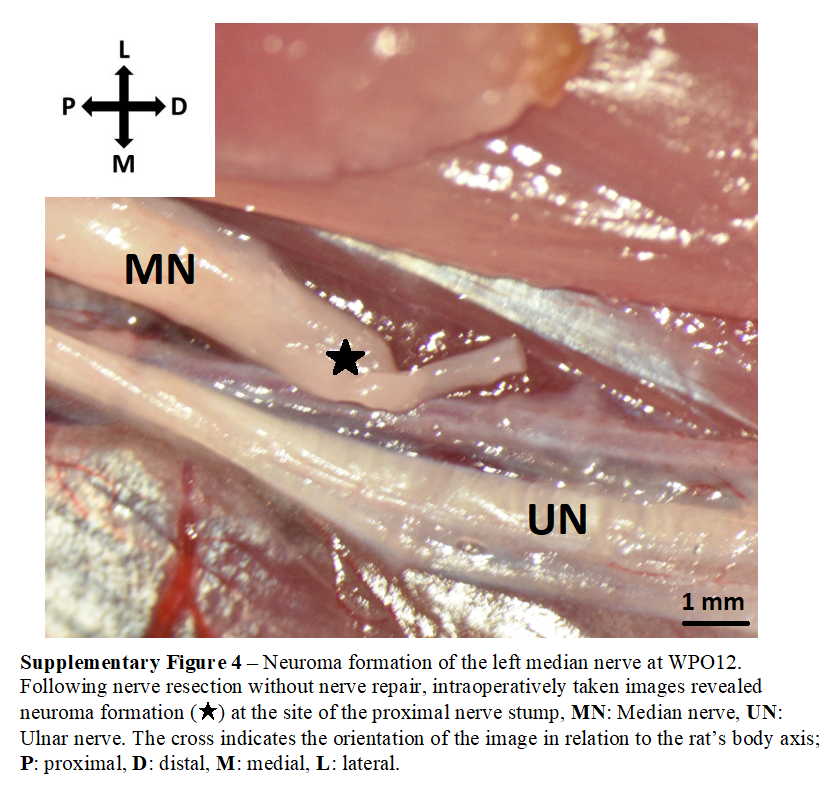

Supplement: Supplementary file 8 [file Image_4.TIFF]
